# Leadership Competencies of the Medical-Surgical Nursing Specialist Nurse

**DOI:** 10.1590/0034-7167-2022-0721

**Published:** 2023-12-04

**Authors:** Marina Filipa Silveira Pires, Romana Silva Lopes, Cláudia Sofia Fernandes Caetano, Liliana Andreia Neves da Mota, Fernanda Maria Príncipe Bastos Ferreira

**Affiliations:** I Hospital de Santo Espírito da Ilha Terceira. Angra do Heroísmo, Açores, Portugal; II Centro Hospitalar e Universitário de Coimbra. Coimbra, Portugal; III Escola Superior de Saúde Norte da Cruz Vermelha

**Keywords:** Nursing, Medical-Surgical Nursing, Skills, Leadership, Self-Perception, Enfermería, Enfermería Medico-Quirúrgica, Habilidades, Liderazgo, Autopercepción, Enfermagem, Enfermagem Médico-Cirúrgica, Competências, Liderança, Autoperceção

## Abstract

**Objective:**

To identify the leadership competencies of Medical-Surgical Nursing Specialist Nurses.

**Methods:**

A quantitative, descriptive study using the Leadership Competencies Questionnaire. 311 Portuguese Medical-Surgical Nursing Specialist Nurses participated. Data analysis involved descriptive and inferential statistical analysis using the Statistical Package for Social Sciences (SPSS), version 22.0.

**Results:**

Study participants had an above-average self-perception on the scale (mean = 3.5) in all leadership roles, recognizing their leadership competencies. The leadership competencies of Medical-Surgical Nursing Specialist Nurses are balanced across all roles: Mentor (5.80 ± 1.02); Coordinator (5.53 ± 0.86); Facilitator (5.38 ± 1.04); Innovator (5.34 ± 0.88); Director (5.31 ± 1.10); Producer (5.30 ± 0.98); Monitor (5.15 ± 1.00); Corrector (4.79 ± 1.29)

**Conclusions:**

Specialized nursing practice enhances nurses’ self-perceived leadership competencies. Nurses see themselves as leaders focused on internal support and facilitation of collective effort and opportunities for skill development.

## RESUMO

**Objetivo:**

Identificar as competências de liderança dos Enfermeiros Especialistas em Enfermagem Médico-Cirúrgica.

**Métodos:**

Estudo quantitativo, descritivo, utilizando o Questionário de Competências de Liderança. Participaram 311 Enfermeiros Especialistas em Enfermagem Médico-Cirúrgica portugueses. Na análise de dados, recorreu-se à análise estatística descritiva e inferencial através do programa Statistical Package for Social Sciences (SPSS), versão 22.0.

**Resultados:**

Os participantes do estudo têm uma autopercepção acima da média da escala (média=3,5) em todos os papéis de liderança, reconhecendo suas competências de liderança. As competências de liderança dos Enfermeiros Especialistas em Enfermagem Médico-Cirúrgica estão equilibradas entre todos os papéis: Mentor (5,80±1,02), Coordenador (5,53±0,86), Facilitador (5,38±1,04), Inovador (5,34±0,88), Diretor (5,31±1,10), Produtor (5,30±0,98), Monitor (5,15±1,00), Corretor (4,79±1,29).

**Conclusões:**

A prática de uma enfermagem especializada aumenta a percepção das competências de liderança do enfermeiro sobre si próprio. Os Enfermeiros veem-se assim como líderes voltados para uma orientação interna de apoio do esforço coletivo e facilitador de oportunidades e desenvolvimento de competências.

## RESUMEN

**Objetivo:**

Identificar las competencias de liderazgo de los Enfermeros Especialistas en Enfermería Médico-Quirúrgica.

**Métodos:**

Estudio cuantitativo, descriptivo, utilizando el Cuestionario de Competencias de Liderazgo. Participaron 311 Enfermeros Especialistas en Enfermería Médico-Quirúrgica portugueses. En el análisis de datos, se utilizó el análisis estadístico descriptivo e inferencial a través del programa Statistical Package for Social Sciences (SPSS), versión 22.0.

**Resultados:**

Los participantes en el estudio tienen una autoevaluación por encima del promedio en la escala (media=3,5) en todos los roles de liderazgo, reconociendo sus competencias de liderazgo. Las competencias de liderazgo de los Enfermeros Especialistas en Enfermería Médico-Quirúrgica están equilibradas en todos los roles: Mentor (5,80±1,02), Coordinador (5,53±0,86), Facilitador (5,38±1,04), Innovador (5,34±0,88), Director (5,31±1,10), Productor (5,30±0,98), Monitor (5,15±1,00), Corrector (4,79±1,29).

**Conclusiones:**

La práctica de la enfermería especializada aumenta la percepción de las competencias de liderazgo del enfermero sobre sí mismo. Los Enfermeros se ven a sí mismos como líderes orientados hacia una orientación interna de apoyo al esfuerzo colectivo y facilitadores de oportunidades y desarrollo de competencias.

## INTRODUCTION

Nursing is a continuously evolving science in which the care needs and their delivery are becoming increasingly complex. Excellence in practice is demanded, involving the provision of complex evidence-based care and the making of intricate decisions. In this context, it becomes essential to develop leaders within healthcare organizations, adopt leadership behaviors, and acquire the ability to build competencies for guiding individuals from a multidisciplinary team towards achieving specific goals or objectives effectively^(1-2)^.

Leadership in healthcare settings must be considered in a comprehensive manner, taking into account the selection of professionals who will significantly contribute to the improvement of these services. In healthcare organizations, leadership holds extreme importance due to its impact on healthcare outcomes^(3)^. However, many nurses function merely as supervisors and do not attain the level of leadership. Nurses often do not feel fully competent, particularly due to issues related to evidence-based practice, with workplace culture factors inhibiting nursing leadership^(4)^.

The leadership skills of a nurse leader are part of advanced practice, with superior performances leading to an enhancement in the quality and safety of healthcare delivery^(5-6)^. This is, therefore, a competency that requires extensive knowledge, skills, and attitudes to ensure that objectives are met through inspirational people management. It is the ability to bring out the best in each team member towards the organization’s expected results^(7)^. Hence, leadership is understood as an important competency that goes beyond a mere position, where self-awareness is compared to a crucible. That is to say, only those who truly know themselves can be effective leaders^(8-9)^.

In the healthcare sector, nursing is currently

the professional class with the greatest representation, thus necessitating the development of leaders capable of making decisions for the individual and collective well-being, as well as establishing relationships among team members to reconcile individual needs with organizational ones^(10-12)^.

The Portuguese Order of Nurses^(13)^, in its work in this field, views the specialist nurse as a fundamental element in advanced nursing practice, as they possess in-depth knowledge in a specific nursing domain, taking into account human responses to life processes and health problems. They demonstrate high levels of clinical judgment and decision-making, reflected in a set of specialized competencies related to an area of intervention, in which advanced nursing practice involves the use and development of guidance and leadership skills.

This study aims to contribute by deepening the competencies identified during the leadership process, which are effective and help leaders recognize their strengths and weaknesses, thus contributing to the delivery of excellent nursing care.

## OBJECTIVE

To identify the leadership competencies of Medical-Surgical Nursing Specialist Nurses.

## METHODS

### Ethical Considerations

The study adhered to national and international ethical guidelines and received approval from the Research Ethics Committee of the Portuguese Red Cross Northern School, with their opinion attached to this submission. Participation in the study was voluntary, confidentiality was assured, and participants had the option to withdraw at any time without repercussions. Informed consent was electronically obtained from all individuals involved in the study.

### Study Design, Period, and Location

This is a quantitative descriptive study, conducted following the Strengthening the Reporting of Observational Studies in Epidemiology (STROBE) guidelines for reporting observational studies. The questionnaire was administered electronically and sent via email in collaboration with the Order of Nurses to all Medical-Surgical Nursing Specialist Nurses in Portugal from August 27, 2021, to September 10, 2021.

### Population and Sample

A total of 311 Medical-Surgical Nursing Specialist Nurses participated in the study, selected through non-probabilistic convenience sampling. Inclusion criteria required holding the title of Medical-Surgical Nursing Specialist Nurse recognized by the Portuguese Order of Nurses and practicing the profession within Portuguese territory. Excluded were nurses in general care, nurses specializing in rehabilitation nursing, maternal and obstetric nursing, mental health and psychiatric nursing, community health nursing, and child and psychiatric health nursing.

### Study Protocol

Data were collected using a self-assessment questionnaire based on the Leadership Competencies Questionnaire adapted by Parreira^(14)^ for the Portuguese population, referencing Quinn from 1995 and the instrument he developed. The internal consistency values (Cronbach’s alpha) of the Leadership Competencies Questionnaire ranged from 0.86 to 0.94. In this study, the Cronbach’s alpha for the dimensions of leadership competencies is 0.97. The data collection instrument comprises two parts: Part 1 - sociodemographic variables, and Part 2 - Leadership Competencies Questionnaire. Sociodemographic variables include age, gender, years of education, academic qualifications, years of professional experience as a nurse, and years of professional experience as a Medical-Surgical Nursing Specialist Nurse. The Leadership Competencies Questionnaire consists of 32 questions distributed across 8 dimensions: Mentor (items: 8, 16, 20, 29), Facilitator (items: 5, 11, 24, 31), Corrector (items: 3, 13, 18, 27), Innovator (items: 1, 10, 22, 25), Monitor (items: 4, 14, 17, 32), Coordinator (items: 2, 9, 21, 28), Director (items: 7, 12, 19, 26), and Producer (items: 5, 15, 23, 30). The 32 questions were organized following Likert scale criteria, with 7 response options and scores ranging from 1 to 7 (“almost never” - 1 point, “very rarely” - 2 points, “rarely” - 3 points, “occasionally” - 4 points, “frequently” - 5 points, “very frequently” - 6 points, and “almost always” - 7 points). Each of the 32 questionnaire questions corresponds to a behavior reflecting a specific leadership role. The score for each of the dimensions was calculated based on the mean, excluding null values for each item within each dimension.

### Analysis of Results and Statistics

Data processing was conducted using the Statistical Package for the Social Sciences (SPSS), version 22.0, which included descriptive analysis of the data and inferential approaches. Data normality was assessed using the Shapiro-Wilk test. Scores for each leadership role were correlated using Pearson’s correlation coefficient. The level of significance was set at p < 0.05.

## RESULTS

The participants are predominantly female, making up 75.9% (n=236) of the sample. The average age of the participants is 43.09 years (±8.612), with an age range from 27 to 64 years. Regarding their years of nursing experience, participants have an average of 20.00 years (±8.34) of professional practice, with experience ranging from 6 to 44 years. As for their tenure as specialist nurses, the average is 7.44 years (±6.65), spanning from 0 to 32 years.

In terms of educational qualifications, 58.5% (n=182) of the participants hold a master’s degree, 39.5% (n=123) possess a bachelor’s degree, and 1.9% (n=6) have earned a doctorate. Notably, study participants have a self-perception above the scale average (mean=3.5) in all leadership roles, indicating their recognition of their leadership competencies.

It’s worth mentioning that the roles receiving the most attention are Mentor, Facilitator, and Innovator. Corrector, Monitor, Producer, and Director have a lower average leadership score (Table 1).

**Table 1:** Descriptive statistics of leadership roles for the Medical-Surgical Nursing Specialist Nurse

Roles	Minimum	Maximum	Mean	Standard Deviation
Facilitator	1.75	7	5.38	1.04
Mentor	1.75	7	5.80	1.02
Innovator	2.25	7	5.34	0.88
Corrector	1.00	7	4.79	1.29
Director	1.75	7	5.31	1.10
Coordinator	2.00	7	5.53	0.86
Monitor	1.50	7	5.15	1.00
Producer	1.25	7	5.30	0.98

Using the Pearson correlation coefficient (r), it was observed that the leadership roles exhibit positive and significant correlations with each other (Table 2).

**Table 2:** Correlation matrix among leadership roles regarding the Leadership Competencies of the Medical-Surgical Nursing Specialist Nurse

	Facilitator	Mentor	Innovator	Corrector	Producer	Director	Coordinator	Monitor
Facilitator	1	0.73^**^	0.78^**^	0.70^**^	0.85^**^	0.87^**^	0.78^**^	0.76^**^
Mentor		1	0.55^**^	0.53^**^	0.70^**^	0.74^**^0	0.73^**^	0.59^**^
Innovator			1	0.68^**^	0.77^**^	0.76^**^	0.69^**^	0.70^**^
Corrector				1	0.70^**^	0.75^**^	0.68^**^	0.63^**^
Producer					1	0.87^**^	0.79^**^	0.82^**^
Director						1	0.79^**^	0.80^**^
Coordinator							1	0.67^**^
Monitor								1

Note:

** Significance level p<0.001 for all correlations.

We can group the leadership roles into four quadrants, divided by the axes of Flexibility/Control and Internal Orientation/External Orientation, as outlined by Quinn’s Model. Keeping this in mind, the positions of the Medical-Surgical Nursing Specialist Nurses were calculated in relation to the axes predefined by Quinn, based on their self-perceived leadership competencies. The calculation of these variables was done based on the mean, disregarding null values of the variables that compose them.

Participants appear to perceive themselves as more internally oriented, with an average of 5.46 (±0.86), as opposed to external orientation, which has a lower average of 5.18 (±0.96). Regarding the flexibility/control axis, the obtained values were very similar.

It was observed that there are no statistically significant differences between the leadership roles of the Medical-Surgical Nursing Specialist Nurse and the sociodemographic variables used in the study.

## DISCUSSION

The Medical-Surgical Nursing Specialist Nurse, in their practice of advanced nursing, must recognize their leadership competencies in order to mobilize them in the various care situations required. In this study, we observed that the self-perception of leadership competencies among Medical-Surgical Nursing Specialist Nurses is balanced across all roles.

The participants are predominantly female, reflecting the historical nature of nursing, which has traditionally been predominantly composed of women^(15)^.

The quadrant emphasizing interaction and the human process stands out. In this quadrant, the following roles are defined: the Facilitator, who encourages the expression of opinions, seeks consensus, and negotiates compromises; and the Mentor, who, aware of individual needs, promotes active listening, acts fairly, supports legitimate requests, and seeks to facilitate individual development^(14)^. We observed that the Mentor role reflects the highest level of self-perception among nurses, who see themselves as “promoters of people’s development through empathetic guidance, facilitating opportunities for training and skill development”^(14)^.

According to Schmidt^(2)^, a “mentor” leader emphasizes the sensitivity of people, striving to achieve goals fairly and openly, and advocating for the building of skills and human resources.

Next is the Coordinator role, representing the leader responsible for maintaining the structure and flow of the system in continuous operation, safeguarding and minimizing dysfunction and conflicts. The third most perceived role by Medical-Surgical Nursing Specialist Nurses was the Facilitator, who acts as a leader promoting collective effort, creating cohesion, unity, and group spirit^(14)^.

The lower-left quadrant reflects the internal process of the structure, focusing on internal control and stability. It specifies two leadership functions: the Coordinator, who maintains the structure, sets schedules, coordinates, resolves problems, and ensures compliance with rules and standards; and the Monitor, who collects and disseminates information, assesses performance, and provides a sense of continuity and stability^(3)^.

In this study, we observed that the self-perception of leadership competencies is predominantly oriented towards internal orientation, especially in the models of Human Relations and Internal Processes. This orientation is characterized by leaders who promote collective effort, guide and support their colleagues empathetically, but also ensure that people comply with defined rules and objectives^(3)^.

Supporting Melo’s study^(16)^, the human relations model scored the highest, indicating a greater need for leaders with the ability to manage conflicts, create cohesion among team members, establish interpersonal communication, and develop their subordinates.

On the other hand, the study reveals that in the external orientation axis, Medical-Surgical Nursing Specialist Nurses identify less. The upper-right quadrant is related to Open Systems Theory and the organization’s adaptation process to the external environment, defining two leadership functions: the Innovator, who is creative, predictive, encouraging, and a facilitator of change; and the Corrector, who is politically astute, acquires resources, and maintains the external legitimacy of the unit through the development and maintenance of external contacts^(3)^. It was the Corrector role that Medical-Surgical Nursing Specialist Nurses identified with the least, seeing themselves as having fewer competencies to represent a leader who is “politically astute, persuasive, influential, and powerful in promoting external legitimacy and obtaining the necessary resources”^(14)^.

In contrast, in the lower-right quadrant, two more leadership functions are specified based on meeting external goals. Leadership is focused on defining and motivating the achievement of these goals. These roles are as follows: the Producer, who is oriented towards task and work fulfillment, motivating behaviors that enable its implementation; and the Director, who is involved in goal setting, role clarification, objective definition, and setting clear expectations^(3)^.

**Figure 1:**
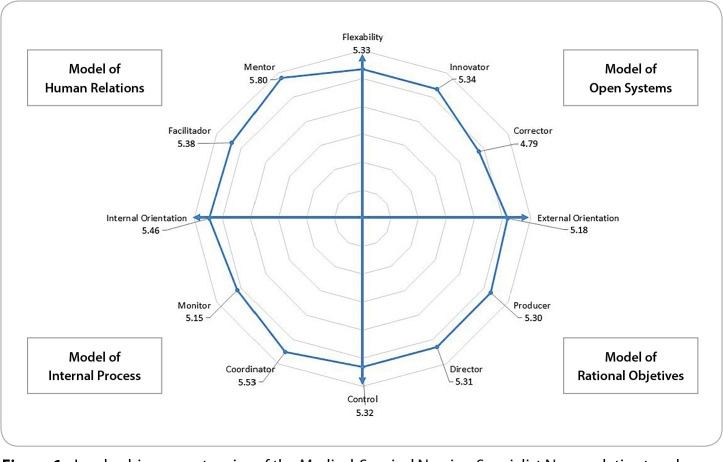
Leadership competencies of the Medical-Surgical Nursing Specialist Nurse relative to roles

In this study, we observed that there is a lower perception of their own leadership competencies oriented towards external orientation, mainly in the Open Systems and Rational Goal models (Figure 1). Thus, Medical-Surgical Nursing Specialist Nurses do not perceive themselves as influential and persuasive, considering themselves less capable of obtaining the necessary resources to make the necessary changes^(3)^.

In the study conducted by Mota^(3)^, which characterizes the leadership roles of head nurses by subordinate nurses, we can see that all leadership roles are related to each other. In the mentioned study, the Mentor role was the most prominent, as also observed in our study. Additionally, the roles with a greater focus demonstrate an orientation towards control and internal orientation, which corroborates the results of our study.

Nursing is a profession that requires effective leaders developed in all leadership roles, resulting in higher levels of performance and, consequently, professional satisfaction^(16)^. We suggest that further studies be conducted in this area to understand the importance of the role of Medical-Surgical Nursing Specialist Nurses in changing the paradigm of nursing in Portugal.

### Study Limitations

This research has limitations that should be considered. The limited time for data collection may have impacted the size of the final sample. A small sample size can affect the representativeness of the results and limit the generalizability of the conclusions. Secondly, our research is primarily based on descriptive analyses and correlations, which also make it challenging to generalize the findings. Finally, the inclusion criterion of specialist nurses in Medical-Surgical Nursing may introduce selection bias, as nurses with this specialization may have specific characteristics or experiences that differentiate them from other nurses.

### Contributions to Nursing, Health, and Public Policies

Medical-Surgical Nursing Specialist Nurses need to recognize and develop their leadership competencies, contributing to their personal and professional development, which can result in better individual, team, and institutional performance in the future. We believe that a change in the mindset and vision of nursing is necessary to achieve higher levels of professional satisfaction among all nurses, where the competencies and characteristics of a leader can be developed throughout life.

## CONCLUSIONS

The practice of specialized nursing increases the perception of a nurse’s self-leadership competencies. Medical-Surgical Nursing Specialist Nurses see themselves as leaders oriented towards internal guidance, supporting collective effort and facilitating opportunities and skill development, while adhering to defined rules and objectives, as mentioned in the Human Relations and Internal Processes models. On the other hand, it was observed that there is a lower perception of their own leadership competencies oriented towards external orientation, according to the Open Systems and Rational Goal models, where Medical-Surgical Nursing Specialist Nurses see themselves as less influential and persuasive, lacking external influence, and considering themselves less capable of obtaining the necessary resources to make the necessary changes. Promoting an outward-oriented perspective leads to increased benchmarking, thus promoting the improvement of care quality. In responding to our research question, we consider it important to continue the development of evidence-based practice and invest in the training of Medical-Surgical Nursing Specialists so that we can be active actors in improving excellent nursing care.

## Supplementary Material

0034-7167-reben-76-06-e20220721-suppl01

0034-7167-reben-76-06-e20220721-suppl02
