# Supplementary material for: Leadership Competencies of the Medical-Surgical Nursing Specialist Nurse
Source: Rev Bras Enferm. 2023 Dec 4;76(6):e20220721. doi: 10.1590/0034-7167-2022-0721 (PMC10695035; doi:10.1590/0034-7167-2022-0721)
Supplement: 0034-7167-reben-76-06-e20220721-suppl01 [file 0034-7167-reben-76-06-e20220721-suppl01.pdf]

Autopercepção das competências de liderança do Enfermeiro Especialista em Enfermagem Médico-Cirúrgica

As enfermeiras Cláudia Caetano, Marina Pires e Romana Lopes do 13º Curso de Pós-Licenciatura de Especialização em Enfermagem Médico-Cirúrgica da Escola Superior de Saúde Norte da Cruz Vermelha Portuguesa, encontram-se a realizar um estudo no âmbito da unidade curricular de Investigação em Enfermagem, sob a orientação da Professora Liliana Mota e da Professora Fernanda Príncipe.

O objetivo do estudo é analisar e demonstrar a autopercepção do Enfermeiro Especialista em Enfermagem Médico-Cirúrgica relativamente às suas competências de liderança em Enfermagem.

Para atingir o nosso objetivo realizámos um questionário para aplicar a todos os enfermeiros com o título de Especialista em Enfermagem Médico-Cirúrgica com inscrição ativa na Ordem dos Enfermeiros, e a exercer em território Português. Por outro lado o critério de exclusão é não ter o título ativo na Ordem dos Enfermeiros e exercer fora do território Português.

O questionário é composto por duas partes: caraterização sociodemográfica e Questionário de Competências de Liderança (QCL), adaptado de Parreira et al. (2006).

Todas as informações e dados recolhidos são confidenciais e anónimos. As respostas cedidas serão objeto de tratamento agregado, sendo que serão somente utilizadas para fins académicos.

Ao responder ás seguintes perguntas está a dar o seu consentimento explícito para a utilização dos dados para fins de investigação.

Agradecemos especial atenção para não colocar dados pessoais que permitem a sua identificação, em qualquer parte do questionário.

Disponibilizamo-nos para qualquer informação adicional através do email do investigador principal: marina_pires16@hotmail.com.

Agradecemos a sua participação e disponibilidade.

*Obrigatório

Caracterização sociodemográfica

1. Idade: *

____

2. Género *

Marcar apenas uma oval.

- Feminino
- Masculino

3. Anos de escolaridade *

____

4. Habilitações literárias *

Marcar apenas uma oval.

- Licencitatura
- Mestrado
- Doutoramento

5. Tempo de experiência profissional como Enfermeiro *

____

6. De momento, possui o título de Enfermeiro Especialista em Enfermagem Médico-Cirúrgica? *

Marcar apenas uma oval.

- Sim
- Não

7. Tempo de experiência profissional como Enfermeiro Especialista em Enfermagem Médico-Cirúrgica *

____

8. De momento, encontra-se a exercer funções em Portugal? *

Marcar apenas uma oval.

- Sim
- Não

Questionário de Competências de Liderança (QCL)

Pense na sua forma de agir, no seu dia-a-dia. Diga-nos com que frequência apresenta os seguintes comportamentos.
1 - Quase nunca; 2 - Muito raramente; 3 - Raramente; 4 - Ocasionalmente; 5 - Frequentemente; 6 - Muito frequentemente; 7 - Quase sempre.

9. 1 - Propõe ideias criativas:

Marcar apenas uma oval.

1	2	3	4	5	6	7

10. 2 - Promove a continuidade das operações diárias na unidade:

Marcar apenas uma oval.

1	2	3	4	5	6	7

11. 3 - Exerce influência nos níveis mais altos da organização:

Marcar apenas uma oval.

1	2	3	4	5	6	7

12. 4 - Revê cuidadosa e detalhadamente toda a informação disponível:

Marcar apenas uma oval.

1	2	3	4	5	6	7
⬭	⬭	⬭	⬭	⬭	⬭	⬭

13. 5 - Mantém na unidade uma orientação para os resultados:

Marcar apenas uma oval.

1	2	3	4	5	6	7
⬭	⬭	⬭	⬭	⬭	⬭	⬭

14. 6 - Facilita a construção de consensos no trabalho da unidade:

Marcar apenas uma oval.

1	2	3	4	5	6	7
⬭	⬭	⬭	⬭	⬭	⬭	⬭

15. 7 - Define áreas de responsabilidade para os subordinados:

Marcar apenas uma oval.

1	2	3	4	5	6	7
⬭	⬭	⬭	⬭	⬭	⬭	⬭

16. 8 - Ouve os problemas pessoais dos subordinados:

Marcar apenas uma oval.

1	2	3	4	5	6	7
◯	◯	◯	◯	◯	◯	◯

17. 9 - Minimiza ruturas no fluxo de trabalho:

Marcar apenas uma oval.

1	2	3	4	5	6	7
◯	◯	◯	◯	◯	◯	◯

18. 10 - Utiliza/experimenta conceitos e procedimentos novos:

Marcar apenas uma oval.

1	2	3	4	5	6	7
◯	◯	◯	◯	◯	◯	◯

19. 11 - Encoraja a tomada de decisão participativa:

Marcar apenas uma oval.

1	2	3	4	5	6	7
◯	◯	◯	◯	◯	◯	◯

20. 12 - Assegura que todos saibam os objetivos da unidade:

Marcar apenas uma oval.

1	2	3	4	5	6	7
◯	◯	◯	◯	◯	◯	◯

21. 13 - Influência decisões tomadas em níveis superiores:

Marcar apenas uma oval.

1	2	3	4	5	6	7
◯	◯	◯	◯	◯	◯	◯

22. 14 - Compara registos e relatórios na procura de discrepâncias:

Marcar apenas uma oval.

1	2	3	4	5	6	7
◯	◯	◯	◯	◯	◯	◯

23. 15 - Verifica se são cumpridos os objetivos estabelecidos:

Marcar apenas uma oval.

1	2	3	4	5	6	7
◯	◯	◯	◯	◯	◯	◯

24. 16 - Demonstra empatia e preocupação na relação com os subordinados:

Marcar apenas uma oval.

1	2	3	4	5	6	7
⬭	⬭	⬭	⬭	⬭	⬭	⬭

25. 17 - Trabalha com informação técnica

Marcar apenas uma oval.

1	2	3	4	5	6	7
⬭	⬭	⬭	⬭	⬭	⬭	⬭

26. 18 - Tem acesso a pessoas de níveis mais elevados:

Marcar apenas uma oval.

1	2	3	4	5	6	7
⬭	⬭	⬭	⬭	⬭	⬭	⬭

27. 19 - Clarifica objetivos da unidade de trabalho:

Marcar apenas uma oval.

1	2	3	4	5	6	7
⬭	⬭	⬭	⬭	⬭	⬭	⬭

28. 20 - Trata as pessoas de modo delicado e cuidado:

Marcar apenas uma oval.

1	2	3	4	5	6	7
◯	◯	◯	◯	◯	◯	◯

29. 21 - Mantém as rédeas do que se passa na unidade:

Marcar apenas uma oval.

1	2	3	4	5	6	7
◯	◯	◯	◯	◯	◯	◯

30. 22 - Resolve problemas com criatividade:

Marcar apenas uma oval.

1	2	3	4	5	6	7
◯	◯	◯	◯	◯	◯	◯

31. 23 - Impulsiona a unidade para o cumprimento dos seus objetivos:

Marcar apenas uma oval.

1	2	3	4	5	6	7
◯	◯	◯	◯	◯	◯	◯

32. 24 - Encoraja os subordinados a partilhar ideias em grupo:

Marcar apenas uma oval.

1	2	3	4	5	6	7
⬭	⬭	⬭	⬭	⬭	⬭	⬭

33. 25 - Procura inovações e melhorias potenciais:

Marcar apenas uma oval.

1	2	3	4	5	6	7
⬭	⬭	⬭	⬭	⬭	⬭	⬭

34. 26 - Clarifica prioridades e melhorias potenciais:

Marcar apenas uma oval.

1	2	3	4	5	6	7
⬭	⬭	⬭	⬭	⬭	⬭	⬭

35. 27 - Apresenta aos superiores hierárquicos ideias convincentes:

Marcar apenas uma oval.

1	2	3	4	5	6	7
⬭	⬭	⬭	⬭	⬭	⬭	⬭

36. 28 - Traz à unidade uma sensação de ordem:

Marcar apenas uma oval.

1	2	3	4	5	6	7
◯	◯	◯	◯	◯	◯	◯

37. 29 - Preocupa-se com as necessidades dos subordinados:

Marcar apenas uma oval.

1	2	3	4	5	6	7
◯	◯	◯	◯	◯	◯	◯

38. 30 - Realça a obtenção das propostas previstas:

Marcar apenas uma oval.

1	2	3	4	5	6	7
◯	◯	◯	◯	◯	◯	◯

39. 31 - Cria uma equipa de trabalho com grupos:

Marcar apenas uma oval.

1	2	3	4	5	6	7
◯	◯	◯	◯	◯	◯	◯

40. 32 - Analisa planos e projetos:

Marcar apenas uma oval.

1	2	3	4	5	6	7
◯	◯	◯	◯	◯	◯	◯

Obrigado!

Este conteúdo não foi criado nem aprovado pela Google.

Google Formulários
