# Supplementary material for: Leadership Competencies of the Medical-Surgical Nursing Specialist Nurse
Source: Rev Bras Enferm. 2023 Dec 4;76(6):e20220721. doi: 10.1590/0034-7167-2022-0721 (PMC10695035; doi:10.1590/0034-7167-2022-0721)
Supplement: 0034-7167-reben-76-06-e20220721-suppl02 [file 0034-7167-reben-76-06-e20220721-suppl02.pdf]

Carimbo de data/hora	Pontuação	Idade:	Género
8/2/2021 9:20:15	0	33	Feminino
8/2/2021 9:23:40	0	34	Feminino
8/2/2021 9:30:14	0	41	Feminino
8/2/2021 9:39:01	0	47	Masculino
8/2/2021 9:43:56	0	47	Masculino
8/2/2021 9:50:01	0	33	Feminino
8/2/2021 9:58:05	0	50	Feminino
8/2/2021 10:46:36	0	38	Feminino
8/2/2021 12:00:48	0	38	Masculino
8/2/2021 12:12:21	0	42	Feminino
8/2/2021 12:27:52	0	39	Feminino
8/2/2021 14:09:49	0	46	Feminino
8/2/2021 14:44:18	0	46	Feminino
8/2/2021 19:27:14	0	33	Masculino
8/2/2021 19:52:33	0	43	Feminino
8/2/2021 20:43:06	0	43	Feminino
8/2/2021 21:33:15	0	44	Feminino
8/2/2021 22:34:09	0	42	Masculino
8/2/2021 22:44:23	0	37	Feminino
8/2/2021 23:15:40	0	33	Feminino
8/2/2021 23:40:11	0	42	Masculino
8/3/2021 8:37:57	0	38	Masculino
8/3/2021 10:13:43	0	39	Feminino
8/3/2021 10:17:58	0	44	Masculino
8/3/2021 10:50:53	0	34	Masculino
8/3/2021 20:32:05	0	40	Feminino
8/4/2021 9:52:04	0	35	Masculino
8/4/2021 16:59:32	0	62	Feminino
8/5/2021 14:10:40	0	34	Masculino
8/5/2021 14:12:41	0	35	Masculino
8/5/2021 14:15:27	0	52	Masculino
8/5/2021 14:38:53	0	45	Feminino
8/5/2021 14:53:07	0	37	Feminino
8/5/2021 15:16:48	0	51	Feminino
8/5/2021 15:26:35	0	37	Feminino
8/5/2021 15:27:41	0	34	Feminino
8/5/2021 15:54:29	0	37	Feminino
8/5/2021 16:21:49	0	38	Masculino
8/5/2021 18:10:13	0	33	Feminino
8/5/2021 18:32:28	0	30	Feminino
8/5/2021 21:34:52	0	37	Feminino
8/5/2021 21:48:45	0	35	Masculino
8/6/2021 9:34:14	0	30	Feminino
8/6/2021 10:54:41	0	57	Feminino
8/6/2021 14:10:05	0	37	Feminino
8/6/2021 14:14:40	0	34	Feminino
8/6/2021 14:17:13	0	49	Masculino
8/6/2021 14:29:50	0	35	Feminino

8/6/2021 14:52:45	0	37	Feminino
8/6/2021 15:41:07	0	52	Masculino
8/6/2021 15:41:31	0	39	Masculino
8/6/2021 21:41:25	0	43	Feminino
8/7/2021 9:28:33	0	40	Masculino
8/7/2021 14:03:21	0	36	Feminino
8/7/2021 16:45:35	0	62	Feminino
8/9/2021 21:29:21	0	49	Feminino
8/10/2021 13:23:03	0	34	Feminino
8/11/2021 6:50:53	0	44	Masculino
8/11/2021 8:34:28	0	40	Feminino
8/11/2021 10:56:50	0	36	Masculino
8/11/2021 22:26:08	0	39	Feminino
8/13/2021 7:50:22	0	40	Feminino
8/15/2021 16:24:13	0	61	Masculino
8/15/2021 18:53:15	0	53	Feminino
8/16/2021 14:11:50	0	48	Feminino
8/21/2021 0:06:42	0	38	Masculino
8/21/2021 13:26:40	0	57	Masculino
8/27/2021 11:36:46	0	33	Feminino
8/27/2021 11:37:22	0	30	Feminino
8/27/2021 11:38:33	0	34	Feminino
8/27/2021 11:38:41	0	45	Feminino
8/27/2021 11:39:21	0	39	Feminino
8/27/2021 11:39:56	0	45	Masculino
8/27/2021 11:40:18	0	33	Feminino
8/27/2021 11:40:20	0	32	Feminino
8/27/2021 11:41:44	0	38	Feminino
8/27/2021 11:42:49	0	36	Feminino
8/27/2021 11:43:26	0	39	Masculino
8/27/2021 11:43:49	0	50	Feminino
8/27/2021 11:44:05	0	30	Feminino
8/27/2021 11:45:30	0	47	Feminino
8/27/2021 11:45:32	0	44	Feminino
8/27/2021 11:48:06	0	55	Masculino
8/27/2021 11:48:07	0	37	Masculino
8/27/2021 11:49:55	0	50	Feminino
8/27/2021 11:51:10	0	57	Feminino
8/27/2021 11:51:25	0	51	Feminino
8/27/2021 11:52:58	0	47	Feminino
8/27/2021 11:53:33	0	29	Feminino
8/27/2021 11:54:25	0	32	Feminino
8/27/2021 11:55:55	0	35	Feminino
8/27/2021 11:56:39	0	35	Feminino
8/27/2021 11:58:27	0	27	Masculino
8/27/2021 11:59:41	0	52	Feminino
8/27/2021 12:01:25	0	41	Feminino
8/27/2021 12:02:06	0	59	Feminino
8/27/2021 12:04:03	0	61	Masculino

8/27/2021 12:08:25	0	39	Feminino
8/27/2021 12:08:32	0	53	Masculino
8/27/2021 12:11:37	0	37	Feminino
8/27/2021 12:15:45	0	47	Feminino
8/27/2021 12:16:57	0	34	Feminino
8/27/2021 12:19:37	0	43	Masculino
8/27/2021 12:19:40	0	42	Feminino
8/27/2021 12:20:03	0	52	Masculino
8/27/2021 12:24:32	0	38	Feminino
8/27/2021 12:24:55	0	42	Masculino
8/27/2021 12:26:35	0	43	Feminino
8/27/2021 12:27:52	0	56	Feminino
8/27/2021 12:28:30	0	30	Feminino
8/27/2021 12:28:48	0	30	Feminino
8/27/2021 12:31:12	0	36	Feminino
8/27/2021 12:32:33	0	48	Feminino
8/27/2021 12:33:18	0	40	Feminino
8/27/2021 12:35:48	0	39	Masculino
8/27/2021 12:35:51	0	45	Feminino
8/27/2021 12:37:38	0	33	Feminino
8/27/2021 12:38:01	0	50	Feminino
8/27/2021 12:43:05	0	29	Feminino
8/27/2021 12:50:31	0	44	Feminino
8/27/2021 12:52:35	0	57	Feminino
8/27/2021 12:53:47	0	55	Feminino
8/27/2021 12:53:57	0	40	Feminino
8/27/2021 13:01:32	0	32	Feminino
8/27/2021 13:09:40	0	35	Masculino
8/27/2021 13:10:45	0	56	Masculino
8/27/2021 13:12:12	0	51	Masculino
8/27/2021 13:12:37	0	38	Masculino
8/27/2021 13:15:50	0	57	Feminino
8/27/2021 13:16:42	0	34	Feminino
8/27/2021 13:18:19	0	35	Feminino
8/27/2021 13:21:37	0	59	Masculino
8/27/2021 13:22:17	0	39	Feminino
8/27/2021 13:24:52	0	34	Feminino
8/27/2021 13:34:11	0	29	Feminino
8/27/2021 13:36:29	0	38	Feminino
8/27/2021 13:41:33	0	51	Masculino
8/27/2021 13:43:04	0	40	Masculino
8/27/2021 13:57:44	0	38	Masculino
8/27/2021 13:59:36	0	40	Feminino
8/27/2021 14:06:30	0	38	Feminino
8/27/2021 14:10:09	0	35	Feminino
8/27/2021 14:11:03	0	53	Feminino
8/27/2021 14:13:04	0	31	Masculino
8/27/2021 14:14:34	0	38	Feminino
8/27/2021 14:26:58	0	34	Feminino

8/27/2021 14:28:17	0	47	Feminino
8/27/2021 14:31:26	0	35	Feminino
8/27/2021 14:33:42	0	55	Feminino
8/27/2021 14:39:10	0	47	Masculino
8/27/2021 14:40:10	0	64	Feminino
8/27/2021 14:42:20	0	32	Feminino
8/27/2021 14:42:26	0	51	Feminino
8/27/2021 14:44:14	0	48	Feminino
8/27/2021 14:55:17	0	40	Masculino
8/27/2021 15:00:59	0	41	Feminino
8/27/2021 15:10:24	0	40	Feminino
8/27/2021 15:12:33	0	35	Feminino
8/27/2021 15:13:50	0	58	Feminino
8/27/2021 15:17:02	0	38	Feminino
8/27/2021 15:17:03	0	34	Feminino
8/27/2021 15:19:05	0	41	Feminino
8/27/2021 15:30:31	0	42	Feminino
8/27/2021 15:33:16	0	58	Feminino
8/27/2021 15:39:54	0	30	Feminino
8/27/2021 15:40:43	0	47	Feminino
8/27/2021 15:41:21	0	40	Feminino
8/27/2021 15:43:01	0	60	Feminino
8/27/2021 15:46:21	0	60	Feminino
8/27/2021 15:51:05	0	48	Feminino
8/27/2021 15:52:14	0	41	Feminino
8/27/2021 16:08:05	0	39	Feminino
8/27/2021 16:17:52	0	41	Feminino
8/27/2021 16:23:02	0	37	Masculino
8/27/2021 16:42:04	0	57	Masculino
8/27/2021 16:46:50	0	36	Masculino
8/27/2021 17:04:35	0	47	Masculino
8/27/2021 17:09:30	0	37	Feminino
8/27/2021 17:13:41	0	38	Feminino
8/27/2021 17:24:56	0	39	Feminino
8/27/2021 17:26:50	0	39	Feminino
8/27/2021 17:39:04	0	31	Feminino
8/27/2021 17:48:16	0	59	Feminino
8/27/2021 17:49:48	0	40	Masculino
8/27/2021 17:51:21	0	40	Feminino
8/27/2021 17:59:21	0	34	Feminino
8/27/2021 18:00:03	0	54	Feminino
8/27/2021 18:01:29	0	41	Feminino
8/27/2021 18:03:48	0	60	Masculino
8/27/2021 18:08:10	0	50	Feminino
8/27/2021 18:12:38	0	37	Masculino
8/27/2021 18:18:54	0	37	Masculino
8/27/2021 18:24:50	0	35	Feminino
8/27/2021 19:01:42	0	58	Feminino
8/27/2021 19:15:27	0	43	Feminino

8/27/2021 19:48:09	0	52	Feminino
8/27/2021 19:50:29	0	35	Feminino
8/27/2021 20:17:28	0	41	Feminino
8/27/2021 20:31:09	0	31	Masculino
8/27/2021 20:38:15	0	40	Feminino
8/27/2021 20:38:20	0	34	Feminino
8/27/2021 20:42:08	0	31	Feminino
8/27/2021 20:48:41	0	41	Feminino
8/27/2021 20:59:10	0	51	Feminino
8/27/2021 21:13:06	0	36	Feminino
8/27/2021 22:11:33	0	46	Masculino
8/27/2021 22:51:04	0	38	Feminino
8/27/2021 23:38:16	0	44	Masculino
8/27/2021 23:46:49	0	48	Masculino
8/28/2021 2:56:47	0	32	Feminino
8/28/2021 3:24:56	0	37	Masculino
8/28/2021 5:31:24	0	50	Feminino
8/28/2021 7:17:54	0	48	Masculino
8/28/2021 8:04:52	0	41	Feminino
8/28/2021 8:34:00	0	36	Feminino
8/28/2021 8:39:04	0	52	Masculino
8/28/2021 9:01:12	0	36	Masculino
8/28/2021 9:29:28	0	35	Feminino
8/28/2021 9:33:31	0	39	Feminino
8/28/2021 10:09:12	0	37	Feminino
8/28/2021 10:34:45	0	40	Masculino
8/28/2021 10:35:25	0	46	Feminino
8/28/2021 10:41:18	0	38	Feminino
8/28/2021 10:55:01	0	63	Feminino
8/28/2021 11:06:20	0	38	Feminino
8/28/2021 11:10:07	0	46	Feminino
8/28/2021 11:41:43	0	51	Feminino
8/28/2021 11:59:30	0	57	Feminino
8/28/2021 12:53:45	0	37	Masculino
8/28/2021 13:10:17	0	50	Feminino
8/28/2021 13:44:07	0	39	Feminino
8/28/2021 15:15:36	0	54	Feminino
8/28/2021 15:28:59	0	52	Feminino
8/28/2021 15:51:49	0	47	Feminino
8/28/2021 16:57:58	0	61	Feminino
8/28/2021 18:22:24	0	44	Feminino
8/28/2021 19:41:06	0	58	Feminino
8/28/2021 19:47:56	0	49	Feminino
8/28/2021 19:48:46	0	43	Feminino
8/28/2021 19:52:29	0	33	Feminino
8/28/2021 19:55:32	0	51	Feminino
8/28/2021 20:19:12	0	43	Feminino
8/28/2021 20:53:50	0	53	Feminino
8/28/2021 20:57:57	0	43	Feminino

8/28/2021 21:06:00	0	36	Feminino
8/28/2021 21:08:38	0	53	Feminino
8/28/2021 21:55:05	0	40	Feminino
8/29/2021 0:38:16	0	46	Feminino
8/29/2021 7:57:13	0	38	Masculino
8/29/2021 9:16:30	0	36	Masculino
8/29/2021 9:22:58	0	56	Feminino
8/29/2021 9:56:50	0	44	Feminino
8/29/2021 10:41:51	0	42	Feminino
8/29/2021 13:16:02	0	32	Feminino
8/29/2021 13:57:38	0	43	Feminino
8/29/2021 16:04:07	0	34	Feminino
8/29/2021 17:01:01	0	42	Masculino
8/29/2021 18:04:44	0	58	Feminino
8/29/2021 18:20:12	0	62	Feminino
8/29/2021 19:03:20	0	45	Feminino
8/29/2021 20:01:27	0	34	Feminino
8/29/2021 20:35:23	0	36	Feminino
8/29/2021 20:49:28	0	46	Feminino
8/29/2021 22:44:18	0	37	Feminino
8/30/2021 2:22:22	0	37	Feminino
8/30/2021 9:22:22	0	64	Masculino
8/30/2021 9:31:55	0	46	Masculino
8/30/2021 9:48:25	0	34	Feminino
8/30/2021 9:58:51	0	34	Feminino
8/30/2021 10:15:00	0	39	Feminino
8/30/2021 10:59:57	0	43	Feminino
8/30/2021 11:06:11	0	53	Feminino
8/30/2021 11:15:11	0	53 anos	Masculino
8/30/2021 11:26:00	0	47	Feminino
8/30/2021 12:39:16	0	31	Feminino
8/30/2021 13:22:40	0	56 anos	Feminino
8/30/2021 13:41:43	0	29	Masculino
8/30/2021 14:22:34	0	57	Feminino
8/30/2021 15:38:51	0	54	Masculino
8/30/2021 20:21:36	0	42	Feminino
8/31/2021 8:54:43	0	40	Feminino
8/31/2021 8:55:01	0	43	Feminino
8/31/2021 11:14:08	0	40	Feminino
8/31/2021 11:21:08	0	50	Feminino
8/31/2021 11:31:20	0	50	Feminino
8/31/2021 12:08:48	0	42	Feminino
8/31/2021 12:46:34	0	36	Masculino
8/31/2021 13:09:19	0	45	Feminino
8/31/2021 14:12:58	0	39	Feminino
8/31/2021 15:29:59	0	40	Feminino
8/31/2021 21:32:44	0	42	Feminino
8/31/2021 21:50:36	0	38	Masculino
8/31/2021 21:51:11	0	62	Feminino

9/1/2021 0:05:23	0		39	Feminino
9/1/2021 11:01:14	0		43	Feminino
9/1/2021 12:00:54	0		55	Feminino
9/1/2021 13:22:33	0		35	Masculino
9/1/2021 16:01:28	0		39	Masculino
9/1/2021 16:11:50	0		58	Feminino
9/1/2021 20:11:00	0		41	Feminino
9/1/2021 21:18:41	0		46	Feminino
9/1/2021 22:05:05	0		44	Feminino
9/2/2021 7:15:03	0	57 anos		Feminino
9/2/2021 7:20:59	0		42	Feminino
9/2/2021 18:12:10	0		50	Feminino
9/2/2021 21:06:19	0		51	Feminino
9/3/2021 0:36:04	0		26	Masculino
9/3/2021 5:57:03	0		39	Feminino
9/3/2021 15:23:57	0		52	Feminino
9/3/2021 18:46:33	0		59	Feminino
9/4/2021 4:11:38	0		53	Feminino
9/4/2021 9:39:15	0		47	Feminino
9/5/2021 15:04:22	0		55	Masculino
9/5/2021 22:47:34	0		47	Feminino
9/6/2021 22:44:35	0		45	Feminino
9/7/2021 11:36:22	0		59	Feminino
9/7/2021 14:35:40	0		47	Feminino
9/7/2021 14:52:15	0		40	Feminino
9/7/2021 15:33:21	0		30	Feminino
9/7/2021 17:50:25	0		53	Feminino
9/8/2021 8:20:43	0		57	Feminino
9/9/2021 10:02:26	0		43	Masculino

Anos de escolaridade	Habilitações literárias	Tempo de experiência	De momento, possui o
19 anos	Licencitatura	6 anos	Não
Licenciatura em enferm	Licencitatura	10 anos	Sim
17	Licencitatura	20	Sim
12	Licencitatura	20	Sim
16	Licencitatura	21	Sim
17	Licencitatura	11	Sim
23	Licencitatura	27	Sim
17	Licencitatura	16	Sim
16	Licencitatura	17	Sim
18	Licencitatura	20	Sim
18	Licencitatura	16	Sim
12	Licencitatura	22 anos	Sim
15	Licencitatura	23 anos	Sim
25	Mestrado	9 anos	Sim
12	Licencitatura	21	Sim
12	Licencitatura	22 anos	Sim
18	Licencitatura	14	Sim
12	Licencitatura	18	Sim
19	Licencitatura	14	Sim
19	Mestrado	11	Sim
12	Mestrado	22	Sim
Licenciatura	Mestrado	13	Sim
18	Licencitatura	16	Sim
17	Licencitatura	24	Sim
12	Licencitatura	12	Sim
12	Mestrado	19	Sim
12	Licencitatura	13	Sim
12ano	Mestrado	36 anos	Sim
12	Mestrado	12	Sim
12	Mestrado	14	Sim
12	Mestrado	29 anos 7 meses	Sim
12	Mestrado	25	Sim
20	Mestrado	15	Sim
12ºano	Licencitatura	28 anos	Sim
17	Licencitatura	16	Sim
18	Mestrado	12	Sim
18	Licencitatura	15	Sim
12	Mestrado	17	Sim
Mestrado	Mestrado	12	Sim
18	Mestrado	9	Sim
17	Licencitatura	16	Sim
18	Mestrado	14	Sim
12	Mestrado	6	Sim
17	Mestrado	30	Sim
18	Mestrado	16	Sim
Muitos	Mestrado	12	Não
18	Mestrado	25	Sim
18	Mestrado	12	Sim

	8	Mestrado		14	Sim
	19	Mestrado		25	Sim
	18	Mestrado		17	Sim
Pós graduação		Licencitatura	22 anoa		Sim
	16	Licencitatura		17	Sim
	18	Licencitatura	13anos		Sim
	7	Licencitatura		43	Sim
	12	Mestrado	27 anos		Sim
	16	Licencitatura		10	Sim
	20	Mestrado		21	Sim
	16	Licencitatura		19	Sim
	20	Mestrado		13	Sim
	16	Licencitatura		16	Sim
	20	Mestrado		18	Sim
	12	Doutoramento		36	Sim
Mestrado		Mestrado		32	Sim
	22	Mestrado		27	Sim
	12	Licencitatura		14	Sim
	12	Mestrado		30	Sim
	18	Mestrado	10 anos		Sim
	13	Mestrado		8	Sim
	18	Licencitatura		11	Sim
	12	Licencitatura		24	Sim
Mestrado		Mestrado		16	Sim
	12	Mestrado		22	Sim
?		Mestrado		12	Sim
	12	Mestrado		11	Sim
	16	Licencitatura		16	Sim
12ºano		Mestrado	13anos		Sim
	18	Mestrado		17	Sim
	12	Licencitatura		25	Sim
	20	Mestrado		8	Sim
	12	Licencitatura		26	Sim
	16	Licencitatura		22	Sim
	12	Licencitatura		29	Sim
...		Mestrado	14 anos		Sim
16+2 especialidade		Licencitatura		21	Sim
12 ano		Mestrado		33	Sim
	12	Licencitatura		27	Sim
12 ano		Mestrado		25	Sim
	18	Mestrado		6	Sim
	18	Mestrado		11	Sim
	17	Licencitatura		13	Sim
	19	Mestrado	11 anos		Sim
	12	Licencitatura	6 anos		Sim
	18	Licencitatura		30	Sim
	16	Licencitatura		18	Sim
	12	Mestrado		37	Sim
	12	Licencitatura		29	Sim

	19	Mestrado		18	Sim
12º Ano		Mestrado	29 anos		Sim
	19	Mestrado		16	Sim
	20	Mestrado	24 anos		Sim
	18	Mestrado	12 anos		Sim
	19	Mestrado		20	Sim
	12	Mestrado		16	Sim
	12	Licencitatura		24	Sim
	12	Licencitatura	13 anos		Sim
	21	Mestrado		21	Sim
	18	Mestrado		14	Sim
	12	Mestrado		21	Sim
	18	Mestrado		7	Sim
	12	Mestrado		9	Sim
	16	Mestrado		14	Sim
	12	Licencitatura	26 anos		Sim
12 ano		Mestrado		18	Sim
???		Mestrado		17	Sim
	12	Licencitatura		24	Sim
	19	Mestrado		12	Sim
	12	Licencitatura		27	Sim
	12	Mestrado		7	Sim
Especialidade		Licencitatura		14	Sim
	19	Mestrado		35	Sim
	12	Licencitatura	33 anos		Sim
	18	Mestrado		18	Sim
	18	Mestrado		10	Sim
	18	Mestrado		11	Sim
	12	Licencitatura		34	Sim
	20	Licencitatura		27	Sim
	20	Mestrado		17	Sim
	12	Licencitatura		33	Não
	18	Licencitatura		12	Sim
	12	Mestrado		13	Sim
	17	Licencitatura		37	Sim
Não		Mestrado		13	Sim
	5	Mestrado		13	Sim
Mestrado		Mestrado		7	Sim
	18	Mestrado		14	Sim
	18	Licencitatura		23	Sim
	19	Licencitatura		19	Sim
20 anos		Mestrado	16 anos		Sim
	18	Licencitatura		17	Sim
Ensino superior		Mestrado		16	Sim
	21	Mestrado	13 anos		Sim
12 ano		Mestrado		31	Sim
	18	Licencitatura	9 anos		Sim
Mestrado		Mestrado		14	Sim
	20	Mestrado		12	Sim

19	Mestrado		25	Sim
19	Mestrado	13 anos		Sim
19	Doutoramento	33 anos		Sim
12	Licencitatura		24	Sim
12	Mestrado	33 anos		Sim
19	Mestrado		10	Sim
12	Licencitatura		25	Sim
12	Licencitatura	26 anos		Sim
12	Mestrado		18	Sim
17	Licencitatura		20	Sim
18	Mestrado		16	Sim
.	Mestrado	11 anos		Sim
condições de acesso a	Mestrado	36 anos		Sim
12	Mestrado		17	Sim
12	Mestrado	13 anos		Sim
12	Mestrado		20	Sim
12	Mestrado		22	Sim
12 ano	Licencitatura	37 anos		Sim
Mestrado	Mestrado	8 anos		Sim
12	Mestrado		30	Sim
22	Mestrado		8	Sim
12º	Doutoramento		40	Sim
4	Licencitatura		37	Sim
18	Licencitatura		24	Sim
20	Mestrado	16 anos		Sim
19	Mestrado		18	Sim
19	Mestrado		18	Sim
12	Licencitatura		15	Sim
17	Licencitatura		36	Sim
23	Mestrado		14	Sim
17	Licencitatura		22	Sim
9	Mestrado	13 anos		Sim
18	Mestrado	15 anos		Sim
12 + 6	Licencitatura		18	Sim
Mestrado	Mestrado	15 anos		Sim
20	Mestrado		7	Sim
12	Licencitatura	39 anos		Sim
12	Licencitatura		17	Sim
Mestrado	Mestrado		15	Sim
20	Mestrado		11	Sim
16	Mestrado		33	Sim
18	Mestrado		20	Sim
12	Licencitatura		39	Sim
23	Licencitatura		28	Sim
12ano	Mestrado		16	Sim
17	Mestrado	17 anos		Sim
20	Mestrado		12	Sim
12ºano	Mestrado	33 anos		Sim
Licenciatura	Licencitatura		23	Sim

	12	Licencitatura		27	Sim
Licenciatura		Licencitatura		12	Sim
	20	Mestrado		21	Sim
	18	Mestrado	9 anos		Sim
	18	Mestrado	18 anos		Sim
Mestrado		Mestrado		11	Sim
	25	Mestrado		9	Sim
	19	Mestrado		18	Sim
	12	Licencitatura		27	Sim
	12	Licencitatura		12	Sim
	12	Licencitatura		25	Sim
	18	Licencitatura		16	Sim
	18	Mestrado		21	Sim
	18	Mestrado		27	Sim
	18	Mestrado	10 anos		Sim
	18	Mestrado		15	Sim
12 ano		Mestrado	24 anos		Sim
	12	Mestrado		23	Sim
	18	Mestrado		20	Sim
18?		Licencitatura	13 anos		Sim
	18	Mestrado		29	Sim
	12	Mestrado		16	Sim
	16	Mestrado		13	Sim
	16	Licencitatura	17 anos		Sim
	11	Licencitatura		11	Sim
	20	Mestrado		18	Sim
	17	Licencitatura		25	Sim
	16	Licencitatura		17	Sim
Antigo 7 ano		Mestrado	44 anos		Sim
	17	Licencitatura		16	Sim
	19	Licencitatura		23	Sim
	12	Mestrado		29	Sim
Curso estudos superior		Mestrado	34 anos		Sim
	18	Licencitatura		10	Sim
	12	Mestrado		11	Sim
	19	Mestrado		15	Sim
	21	Doutoramento		6	Sim
	12	Mestrado		30	Sim
19 anos		Mestrado	27 anos		Sim
	12	Licencitatura	36 anos		Sim
	18	Mestrado		21	Sim
	18	Licencitatura		31	Sim
	12	Mestrado		26	Sim
	12	Licencitatura		20	Sim
	18	Mestrado	10 anos		Sim
Ensino superior		Mestrado		27	Sim
	12	Mestrado		21	Sim
	12	Licencitatura		30	Sim
	18	Mestrado		21	Sim

	18	Mestrado	14 anos	Sim
12 ano		Licencitatura	30 anos	Sim
	12	Licencitatura	18	Sim
	12	Licencitatura	24	Sim
	12	Mestrado	16 anos	Sim
	12	Licencitatura	12	Sim
Curso superior		Mestrado	30 anos	Sim
	22	Licencitatura	9anos	Sim
12 + 4 curso + 2 mestra		Mestrado	19 anos	Sim
	20	Mestrado	9	Sim
	22	Licencitatura	22	Sim
	12	Licencitatura	12	Sim
Pos licenciatura		Licencitatura	14	Sim
	17	Mestrado	37 anos	Sim
	12	Licencitatura	41	Sim
	12	Mestrado	24 anos	Sim
	18	Mestrado	12	Sim
	6	Mestrado	13	Sim
	0	Licencitatura	25	Sim
	12	Mestrado	15	Sim
	18	Mestrado	14	Sim
	14	Licencitatura	39	Sim
	20	Mestrado	24	Sim
	18	Mestrado	11,5	Sim
	14	Mestrado	12	Sim
	18	Mestrado	17	Sim
	18	Mestrado	21	Sim
12º ano		Licencitatura	30 anos	Sim
12º ano		Mestrado	26 anos	Sim
	12	Mestrado	23	Sim
12°ano		Mestrado	6 anos	Sim
	20	Mestrado	30	Sim
	6	Mestrado	6	Sim
	12	Mestrado	33	Sim
	19	Licencitatura	31	Sim
	20	Mestrado	17	Sim
	18	Mestrado	20	Sim
	23	Doutoramento	21	Sim
	18	Mestrado	17	Sim
	12	Mestrado	25	Sim
12º ano		Licencitatura	22	Sim
	12	Licencitatura	18	Sim
	30	Mestrado	15	Sim
	19	Mestrado	25	Sim
	18	Licencitatura	14	Sim
	12	Licencitatura	18	Sim
	20	Mestrado	20	Sim
	20	Mestrado	16	Sim
	12	Mestrado	39	Sim

	22	Licencitatura		15	Sim
	12	Mestrado		23	Sim
12 ano		Mestrado	30 anos		Sim
	18	Licencitatura		14	Sim
	16	Licencitatura		18	Sim
	12	Mestrado	33 anos		Sim
12 ano		Licencitatura		18	Sim
Pós Graduaçâo MBA E		Licencitatura	15 anos		Não
	18	Mestrado		23	Sim
12°		Licencitatura	30 anos		Sim
	12	Mestrado		22	Sim
12º		Mestrado	13 anos		Sim
	18	Mestrado		28	Sim
	21	Licencitatura		1	Não
	18	Mestrado		18	Sim
19 anos		Licencitatura		28	Sim
	18	Licencitatura		38	Sim
	12	Licencitatura	30 anos		Sim
	18	Licencitatura		21	Sim
Licenciatura		Licencitatura	31 Anos		Sim
	16	Licencitatura		24	Sim
12º Ano		Mestrado	22 anos		Sim
12 ANO		Doutoramento		34	Sim
	12	Mestrado		25	Sim
	16	Licencitatura		17	Não
	19	Mestrado		7	Sim
	12	Licencitatura		28	Sim
12 ºano		Licencitatura		34	Sim
	15	Mestrado		21	Não

Tempo de experiência	De momento, encontra	1 - Propõe ideias criativ	2 - Promove a continuid
0	Sim	6	6
2 anos a exercer	Sim	4	6
0	Sim	7	7
2,5	Sim	4	7
3	Sim	4	7
6	Sim	6	6
4anos	Sim	4	4
7	Sim	4	4
1	Sim	5	5
8	Sim	5	5
3	Sim	5	5
8 anos	Sim	6	6
8 anos	Sim	4	6
6	Sim	4	7
5	Sim	6	5
3 anos	Sim	4	5
8	Sim	6	6
8 anos	Sim	6	7
7	Sim	4	6
6	Sim	6	6
4	Sim	6	7
13	Sim	5	4
3	Sim	5	5
6	Sim	5	5
3	Sim	5	7
4	Sim	4	7
2	Sim	6	7
10anos	Sim	5	5
4	Sim	6	6
3	Sim	5	5
3 anos	Sim	5	6
7 anos	Sim	7	5
6 anos	Sim	7	7
9 anos	Sim	4	7
1	Sim	3	6
8	Sim	4	7
7	Sim	5	7
3	Sim	7	7
5	Sim	7	6
3 anos	Sim	7	7
1	Sim	3	6
5	Sim	6	6
2	Sim	4	5
25	Sim	6	6
6	Sim	6	7
0	Sim	4	4
4	Sim	4	5
4	Sim	5	5

	3	Sim	5	6
	14	Sim	7	7
3 anos		Sim	5	6
1,5 anos		Sim	4	7
	3	Sim	6	6
2anos		Sim	4	4
	25	Sim	5	6
11 anos		Sim	5	6
	4	Sim	6	7
	10	Sim	5	6
	4	Sim	6	6
	4	Sim	6	6
	3	Sim	5	6
	11	Sim	7	5
	29	Sim	6	6
	8	Sim	5	7
	12	Sim	4	5
	2	Sim	7	6
	10	Sim	4	5
1 ano		Sim	3	3
	4	Sim	6	6
	2	Sim	5	6
	15	Sim	6	7
	5	Sim	4	5
	11	Sim	5	7
	1	Sim	6	7
	3	Sim	4	5
	10	Sim	4	4
4 anos		Sim	4	5
	5	Sim	5	6
	12	Sim	6	6
	3	Sim	5	7
	10	Sim	4	7
	2	Sim	5	5
	10	Sim	6	6
	7	Sim	5	6
	4	Sim	4	5
12 anos		Sim	6	6
	23	Sim	6	6
10 anos		Sim	5	5
	2	Sim	5	5
	5	Sim	6	6
	5	Sim	6	7
5 anos		Sim	5	5
2 anos		Sim	4	6
	12	Sim	5	6
	1	Sim	5	5
	12	Sim	6	7
	16	Sim	5	6

7 meses	Sim	6	6
9 anos	Sim	6	6
4	Sim	6	7
6 anos	Sim	6	7
3 anos	Sim	5	7
10	Sim	5	6
6	Sim	4	6
3	Sim	5	4
4 anos	Sim	6	6
10	Sim	7	7
8	Sim	6	6
9	Sim	6	7
0	Sim	7	7
5	Sim	7	7
0	Sim	5	6
11 anos	Sim	5	6
2	Sim	5	6
7	Sim	6	6
14	Sim	5	7
3	Sim	5	5
14 anos	Sim	5	7
1	Sim	5	5
3	Sim	6	6
27	Sim	7	7
22 anos	Sim	4	5
4	Sim	6	7
1	Sim	3	4
2	Sim	4	7
21	Sim	6	7
8	Não		
11	Sim	5	7
20 anos	Sim	5	5
1 mês	Sim	3	7
3	Sim	4	7
25	Sim	6	6
Não	Sim	5	5
1	Sim	3	7
1 ano	Sim	5	7
1	Sim	6	6
7	Sim	5	5
2	Sim	5	5
7 anos	Sim	6	5
6 anos	Sim	4	5
4	Sim	7	7
3 anos	Sim	4	6
13	Sim	6	6
2 anos	Sim	7	7
3anos	Sim	5	5
5	Sim	5	7

	6	Sim	6	7
3 anos		Sim	5	6
	25	Sim	6	6
	9	Sim	6	7
21 anos 7 meses		Sim	4	5
4 anos		Sim	7	7
	4	Sim	4	7
8 anos		Sim	6	7
	5	Sim	5	5
	0	Sim	6	6
	10	Sim	6	7
4 anos		Sim	6	6
27 anos		Sim	4	7
	9	Sim	4	7
3 anos		Sim	4	7
	6	Sim	4	4
3 A		Sim	5	6
21 anos		Sim	5	6
4 anos		Sim	2	4
	10	Não		
	2	Sim	5	6
	30	Sim	4	6
16 meses		Sim	6	6
	15	Sim	5	7
3 anos		Sim	6	6
	3	Sim	5	7
	10	Sim	3	6
5 anos		Sim	4	7
20 anos		Sim	6	6
	2	Sim	4	5
	4	Sim	5	7
	4	Sim	4	6
Três anos		Sim	5	7
	10	Sim	6	5
1 ano		Sim	4	5
9 meses		Sim	5	7
20 anos		Sim	6	5
	9	Sim	6	6
	10	Sim	6	6
	2	Sim	6	6
	5	Sim	5	5
	11	Sim	6	7
	22	Sim	5	7
9 anos		Sim	5	4
4 anos		Sim	5	6
3 anos		Sim	5	5
	7	Sim	5	6
8 anos		Sim	6	6
	2	Sim	5	6

	3	Sim	4	4
Sim		Sim	5	3
	13	Sim	6	7
4 anos		Sim	7	7
10 anos		Sim	5	7
	6	Sim		7
	3	Sim	7	6
	5	Sim	4	6
	21	Sim	7	6
4 anos		Sim	4	6
	10	Sim	6	7
	2	Sim	5	6
	5	Sim	6	7
	4	Sim	4	5
3 anos		Sim	4	4
	9	Sim	6	6
2 anos		Sim	7	5
	10	Sim	6	7
	4	Sim	5	6
	10	Sim	7	7
	21	Sim	5	5
	0	Sim	5	7
	2	Sim	5	5
Sim		Sim	6	7
	0	Sim	1	7
	10	Sim	5	4
	10	Sim	3	7
	1	Sim	6	6
32 anos		Sim	5	6
	5	Sim	5	4
	8	Sim	6	6
	8	Sim	7	7
23 anos		Sim	5	5
	4	Sim	6	7
	4	Sim	5	5
	2	Sim	6	7
	25	Sim	6	7
12 anos		Sim	6	6
1 ano		Sim	6	6
15 anos		Sim	5	6
	4	Sim	4	7
	18	Sim	4	6
	2	Sim	5	4
	15	Sim	6	7
5 anos		Sim	6	7
	12	Sim	6	5
	3	Sim	5	6
9 anos		Sim	5	5
	9	Sim	4	6

6 meses	Sim	4	6
23anos	Sim	5	7
9 anos	Sim	6	6
14	Sim	6	5
3 anos	Sim	4	5
6	Sim	5	5
8 anos	Sim	4	4
3anos	Sim	6	6
2 anos	Sim	5	7
3	Sim	5	4
15	Sim	4	6
3	Sim	6	6
3	Sim	5	5
26 anos	Sim	6	6
28	Sim	4	7
3 anos	Sim	5	5
não exerço como espe	Sim	4	6
5	Sim	5	6
13	Sim	5	7
3	Sim	6	5
6	Sim	6	6
26	Sim	4	5
8	Sim	7	6
5	Sim	6	7
3	Sim	6	6
5	Sim	6	7
10	Sim	7	7
10 anos	Sim	6	7
6 anos	Sim	5	7
3 anos e 8 meses	Sim	7	7
0	Sim	1	6
8 anos	Sim	4	6
2 anos	Sim	6	6
20	Sim	5	6
5	Sim	5	6
8	Sim	5	7
13	Sim	6	6
11	Sim	6	7
12	Sim	4	6
1	Sim	5	4
14	Sim	6	5
2	Sim	3	5
7	Sim	7	7
10	Sim	5	5
10	Sim	4	4
0	Sim	4	7
10	Sim	6	6
3	Sim	4	5
6	Sim	4	5

	2	Não		
	14	Sim	5	6
11 anos		Sim	5	7
	9	Sim	7	7
	7	Sim	3	6
10 anos		Sim	6	7
	7	Sim	4	7
Atuação por 5 anos em		Não		
	2	Sim	6	6
13 anos		Sim	7	7
10anos		Sim	5	6
14 anos		Sim	5	6
	11	Sim	6	7
	0	Sim	3	5
1ano		Sim	7	7
10 anos		Sim	5	6
	5	Sim	5	7
11 anos		Sim	5	7
	1	Sim	5	6
29 Anos		Sim	6	7
	0	Sim	4	4
5 anos		Sim	5	5
21 ANOS		Sim	7	6
	14	Sim	5	5
	0	Sim	2	2
1 ano		Sim	5	5
	11	Sim	4	6
sim		Sim	5	5
	0	Sim	7	7

3 - Exerce influência nc	4 - Revê cuidadosa e d	5 - Mantém na unidade	6 - Facilita a construçã
5	6	6	6
5	5	4	5
4	5	6	6
1	5	5	5
6	6	7	7
6	4	5	5
2	6	4	4
3	4	4	5
3	5	5	5
1	5	5	5
2	5	3	5
2	6	6	6
4	6	6	7
2	3	3	6
6	5	6	6
5	4	5	4
2	7	4	3
7	6	7	6
1	2	5	5
2	4	3	5
7	7	7	6
3	4	4	5
2	5	3	5
7	4	6	6
6	7	7	7
4	7	6	6
6	5	6	7
6	5	5	5
6	5	6	5
5	6	6	5
5	5	5	6
4	3	5	6
5	6	7	7
3	5	5	5
1	3	2	5
3	7	6	6
4	7	6	7
7	6	5	7
6	6	6	6
7	7	7	7
1	3	2	5
4	5	5	7
5	4	4	5
6	6	7	7
6	7	7	7
1	3	3	3
5	6	5	6
3	5	5	5

6	6	5	5
5	6	5	6
4	7	6	5
3	3	6	6
6	5	6	6
3	4	3	5
6	6	6	5
6	6	6	6
6	5	6	6
3	5	5	6
6	6	6	6
5	7	6	6
5	5	6	6
6	5	6	6
6	6	6	6
5	6	6	7
3	4	4	3
5	6	6	6
1	6	6	6
2	5	4	4
2	5	5	5
2	6	5	4
5	6	7	7
4	5	4	4
5	5	5	5
3	7	7	7
1	3	4	5
4	5	4	3
4	5	5	5
4	4	6	5
4	6	6	6
5	6	6	6
5	5	6	6
3	4	4	4
6	6	7	7
6	6	6	5
1	4	5	5
5	5	6	6
5	6	5	6
5	5	5	5
5	5	5	5
5	6	6	7
7	5	5	6
1	5	7	6
1	6	4	6
4	5	6	6
3	5	5	5
4	6	6	6
4	6	6	7

2	6	6	5
5	5	6	7
4	6	7	6
3	5	6	5
3	6	5	7
1	5	4	4
3	6	6	4
3	7	4	5
7	6	6	6
5	6	7	6
5	7	6	6
5	6	6	7
5	7	7	7
6	7	7	7
5	5	5	5
6	6	6	6
7	6	6	6
6	6	6	6
6	7	6	6
4	6	5	5
6	7	7	5
4	5	4	5
1	6	3	5
5	6	7	7
5	5	5	6
4	5	5	6
2	2	2	1
4	5	5	5
4	7	6	6
4	6	6	6
4	7	6	5
3	4	7	4
4	6	6	5
7	6	6	7
3	4	4	4
1	4	4	3
6	6	6	5
2	5	5	6
4	4	4	4
2	4	5	4
2	7	6	6
1	5	1	2
6	7	7	7
1	6	6	5
2	6	6	7
6	6	7	5
3	3	3	3
5	6	7	6

4	4	7	6
1	6	6	7
5	5	7	7
6	7	7	6
5	5	4	5
6	6	7	7
4	7	4	4
4	7	7	6
4	5	5	3
4	4	6	6
7	6	6	6
4	6	5	5
4	6	6	7
1	7	6	6
7	6	6	6
4	6	5	5
5	5	5	5
4	5	5	5
1	5	4	4
5	6	5	6
4	5	5	5
5	5	5	6
7	7	7	7
2	7	7	7
6	5	6	6
3	6	6	6
5	5	6	7
6	6	6	6
1	4	4	2
6	6	7	7
1	5	5	5
4	7	6	6
6	5	5	6
3	5	5	5
5	6	5	6
6	6	6	6
4	5	5	6
6	6		6
4	5	4	5
5	5	5	6
7	7	7	7
6	5	5	6
2	6	5	4
5	5	4	6
1	4	4	5
2	6	6	6
4	7	6	6
4	6	5	6

3	6	4	4
1	4	5	4
6	6	6	6
7	6	6	7
4	7	7	7
7	7	7	7
5	7	7	6
4	6	5	5
7	6	6	7
6	5	6	6
4	5	7	7
3	6	6	6
6	7	5	6
5	5	4	4
4	4	4	4
3	4	5	5
2	4	4	4
5	6	6	6
4	4	6	4
3	7	7	7
6	5	5	5
3	4	4	5
4	5	5	5
6	6	6	6
1	6	6	5
5	4	6	6
2	3	4	5
6	6	6	6
6	5	6	6
5	6	6	6
6	6	5	4
4	7	7	7
4	6	5	6
2	6	6	6
4	5	5	5
6	6	5	6
7	6	7	6
5	7	7	6
4	5	5	5
5	4	4	5
7	6	6	6
3	4	5	5
2	7	6	7
2	5	5	6
4	6	6	6
5	5	5	6
4	6	6	6
6	6	5	5
5	4	4	5

3	6	3	4
1	6	6	6
2	3	6	7
4	5	5	5
4	6		6
4	5	5	5
2	3	4	5
5	5	4	5
4	4	5	6
4	4	5	4
5	5	4	6
4	6	4	4
4	4	5	5
6	6	6	5
5	6	6	7
5	5	5	5
2	4	4	4
5	5	6	5
5	5	5	4
2	5	4	4
7	6	5	7
4	5	5	5
6	6	7	6
4	5	5	6
4	5	5	6
1	6	5	7
6	5	5	5
4	5	6	6
3	6	6	6
5	5	5	6
2	4	6	5
4	6	6	6
4	5	6	6
3	4	5	6
5	7	5	5
4	5	6	7
7	3	6	6
7	6	7	6
4	6	6	4
3	3	4	5
6	5	6	6
1	4	7	4
7	6	7	7
5	5	6	4
4	5	4	5
5	5	6	6
4	4	5	5
3	5	5	4
5	5	5	5

1	6	6	6
3	6	7	7
5	5	5	7
2	3	4	5
3	5	7	7
2	5	5	5
4	7	6	6
5	6	6	7
6	6	6	6
5	5	6	6
6	7	7	7
1	5	5	4
4	6	5	6
4	5	5	5
7	6	6	6
3	7	4	6
5	6	6	6
6	6	7	7
2	5	5	5
6	5	4	4
6	7	7	7
6	5	6	5
2	3	3	3
4	6	6	5
4	5	5	5
6	5	4	6
7	7	7	7

7 - Define áreas de res	8 - Ouve os problemas	9 - Minimiza ruturas no	10 - Utiliza/experimenta
5	6	6	6
4	4	5	5
5	5	5	6
6	6	6	6
7	7	6	7
6	6	4	3
1	4	3	5
4	5	5	5
4	5	4	5
5	5	5	5
2	3	5	5
5	6	6	6
6	7	7	6
1	5	6	7
4	5	4	5
3	5	6	5
5	7	6	6
6	7	7	5
4	6	6	6
4	5	3	5
7	6	6	7
5	5	5	4
2	3	5	5
6	6	7	5
6	7	5	5
6	7	6	5
3	7	7	6
5	5	5	4
5	6	6	5
7	5	5	5
6	6	6	4
6	6	6	5
	7	7	6
3	5	6	6
1	3	3	2
5	5	5	3
5	7	7	7
5	5	6	5
6	6	6	6
7	7	7	7
1	3	3	2
1	6	6	5
4	3	6	5
7	6	7	6
7	7	7	7
1	4	4	4
5	4	5	5
4	5	5	4

5	5	6	5
4	3	5	5
7	7	6	6
5	6	6	6
7	7	6	6
5	4	4	3
5	7	6	4
6	6	6	6
6	6	5	6
5	6	7	5
6	1	5	6
4	1	6	6
5	3	6	5
5	7	5	5
6	6	6	6
7	6	6	6
3	5	6	4
5	7	6	5
5	5	5	4
4	5	6	5
4	4	4	5
1	1	4	6
7	7	7	6
4	4	4	4
4	5	6	4
5	6	6	6
3	6	6	6
4	3	3	5
4	6	6	5
2	2	4	6
5	6	4	6
6	7	6	5
6	7	5	5
3	3	4	4
6	6	6	6
4	7	6	6
4	5	4	5
	5	5	5
6	6	6	6
4	5	5	5
5	5	5	5
7	7	7	4
6	7	7	6
1	6	6	5
1	1	6	5
6	6	6	5
4	4	4	5
5	2	6	6
6	7	6	6

6	4	4	6
7	6	5	6
4	5	5	6
6	7	6	5
3	6	6	6
1	1	5	3
2	5	5	5
3	5	7	7
6	7	6	6
5	6	5	5
6	7	6	6
7	7	7	6
5	7	7	7
7	7	7	7
3	2	4	6
7	7	7	5
6	6	6	6
6	6	6	6
7	7	7	6
5	5	5	5
6	7	5	6
5	6	5	5
1	7	6	6
7	7	7	6
5	7	6	6
6	6	7	6
1	1	2	2
5	5	1	5
7	6	6	6

5	5	5	6
6	7	6	7
1	1	4	1
5	7	7	5
7	7	6	5
4	4	4	4
1	1	1	4
6	5	4	4
5	4	4	4
5	6	6	5
1	5	3	5
7	7	7	7
1	5	3	1
7	7	7	7
3	4	3	5
6	6	6	6
6	5	6	6
3	3	3	3
6	5	6	6

7	7	6	6
6	5	5	4
7	7	6	6
6	7	7	6
4	7	6	5
7	7	6	7
2	7	7	7
6	6	6	6
5	5	4	5
6	6	6	6
6	6	6	6
6	6	6	6
7	7	7	5
4	4	5	6
6	7	7	7
4	4	4	5
4	4	5	5
6	6	5	5
3	5	4	2
6	5	5	5
6	5	6	4
4	6	6	6
5	7	7	6
7	7	7	7
5	6	6	5
2	7	4	6
7	7	7	5
6	7	6	6
2	6	5	3
6	6	6	6
3	5	6	5
6	6	6	6
5	6	5	
5	5	5	4
5	7	7	7
6	7	4	6
4	6	5	4
6	6	6	5
4	6	5	6
5	5	5	6
6	7	7	6
6	6	7	6
2	6	6	4
6	6	6	5
5	6	5	5
3	4	5	5
6	6	7	7
5	6	3	4

4	7	7	5
1	1	1	4
6	4	6	6
6	7	6	6
5	6	7	7
7	7	7	7
4	6	6	6
5	5	5	4
5	6	6	5
5	4	5	5
5	7	7	7
1	5	6	6
7	7	7	6
4	5	4	4
4	5	4	4
5	5	6	7
3	2	4	5
6	7	6	7
4	5	5	3
1	4	7	7
6	5	5	5
1	1	1	1
4	5	5	4
6	6	6	7
1	7	7	3
6	6	5	4
4	6	6	5
5	6	5	6
6	5	5	6
5	5	5	5
4	5	6	6
5	6	5	6
6	6	5	6
6	6	6	5
4	5	5	5
6	6	5	5
6	6	6	6
6	6	6	6
5	6	6	4
5	5	5	5
7	7	6	5
5	5	5	4
6	6	5	6
6	7	6	4
4	7	6	4
5	6	5	6
6	7	6	6
6	6	4	4
4	3	4	5

2	2	4	4
6	6	6	6
6	7	7	5
4	5	5	6
4	5	5	6
6	5	5	5
3	6	6	6
4	4	4	4
5	4	6	5
5	4	4	5
4	7	6	5
3	4	4	4
5	5	5	5
7	7	5	6
7	7	2	7
6	6	6	6
1	1	3	4
5	6	6	5
4	4	5	4
4	5	5	4
7	7	7	6
5	5	5	4
2	5	5	3
3	3	6	5
5	6	4	4
3	1	5	6
7	7	6	4
7	6	6	5
6	7	6	6
2	7	6	5
2	6	5	7
6	6	6	6
1	1	6	4
4	4	5	5
6	5	6	5
6	7	5	7
1	1	6	7
7	7	6	6
4	4	4	3
	6	6	6
5	5	5	5
1	1	1	4
7	7	5	7
4	6	6	4
3	4	4	4
4	6	5	5
4	5	4	5
4	4	5	3
4	4	5	4

1	1	6	6
7	7	7	7
7	7	6	7
3	7	6	6
6	7	6	6
2	6	5	2

4	5	7	6
7	7	6	7
6	6	6	6
6	7	6	6
7	7	7	7
1	1	4	5
6	6	7	5
5	5	4	5
6	7	7	7
7	7	5	6
4	5	6	5
7	6	6	6
4	4	5	5
5	4	4	4
7	7	7	7
6	6	5	6
2	2	2	2
4	3	6	5
4	6	4	5
5	5	5	4
7	7	7	7

11 - Encoraja a tomada	12 - Assegura que todo	13 - Influência decisões	14 - Compara registos
6	6	6	4
5	4	4	5
6	4	3	2
5	5	4	4
7	7	6	6
5	6	6	3
4	4	1	5
6	6	3	4
5	5	3	3
5	5	2	3
5	5	2	7
6	5	2	5
7	7	6	4
7	5	5	6
6	6	6	3
5	4	5	3
7	6	7	5
7	6	7	5
7	6	6	4
5	4	2	2
7	7	6	6
5	5	2	3
5	5	2	7
6	5	7	4
7	7	6	5
7	6	4	3
6	6	4	1
6	6	6	4
6	6	6	6
7	7	5	6
6	7	5	6
6	7	4	2
7	6	5	6
6	5	3	4
3	3	3	3
6	5	6	6
7	6	6	5
7	5	5	6
7	7	7	7
7	7	7	7
3	3	3	3
7	4	5	1
5	5	4	4
7	7	6	6
7	7	7	7
4	4	4	4
5	6	5	6
5	5	4	3

5	5	4	4
6	5	4	4
6	7	4	3
5	4	4	3
7	7	5	5
4	4	2	3
6	5	6	5
6	6	5	5
6	6	6	5
6	7	3	4
6	6	6	4
6	4	4	5
6	6	6	4
7	6	7	5
7	7	6	6
6	7	5	7
5	5	3	3
7	5	6	3
7	6	1	1
5	5	3	5
5	5	2	5
6	6	1	5
6	7	6	5
5	4	4	4
6	6	6	3
6	5	5	4
6	6	6	6
4	4	4	4
7	5	5	6
4	5	4	4
6	6	6	3
6	5	5	7
7	7	5	4
4	3	3	3
6	6	6	6
4	6	5	6
5	5	4	3
6	6	5	5
6	5	5	5
6	6	5	5
6	5	5	5
7	7	6	6
7	7	6	4
7	5	1	4
5	5	1	4
7	6	6	6
6	6	4	4
7	6	4	4
6	7	4	6

5	5	4	4
7	6	6	5
5	5	6	5
5	6	3	6
6	5	3	3
7	4	1	5
5	4	2	2
6	7	4	2
6	7	6	5
5	6	6	6
7	7	5	6
6	7	5	5
7	5	4	4
7	7	7	7
5	6	4	4
7	7	6	6
6	6	6	4
6	6	6	6
7	7	7	7
5	5	5	5
7	7	6	7
5	5	4	5
6	6	1	4
7	7	6	6
6	6	6	5
7	6	4	3
1	1	1	1
7	5	3	2
6	7	4	6
6	5	4	6
7	6	4	6
3	3	2	1
6	6	4	5
7	7	7	5
4	4	3	3
3	1	1	1
5	5	6	6
4	4	2	2
6	6	4	5
4	2	1	1
6	6	2	7
1	1	1	1
7	7	6	6
5	5	3	1
6	6	6	3
5	3	4	4
4	4	1	1
7	5	5	5

7	6	6	4
5	5	3	3
7	7	5	5
7	6	6	7
7	7	6	2
7	7	6	6
6	4	3	4
6	7	3	6
5	4	4	5
6	6	6	6
7	7	6	6
7	5	4	6
6	7	5	6
7	7	2	5
7	7	7	4
5	5	4	3
5	5	5	5
5	6	4	5
5	4	2	2

5	5	5	5
5	6	4	5
6	6	6	4
6	7	7	7
7	7	7	2
5	6	6	5
6	5	3	3
6	7	5	3
7	7	6	7
4	4	1	2
6	6	5	5
5	4	3	4
6	6	4	6
6	5	6	2
5	5	4	3
7	6	4	4
6	7	5	6
6	4	4	4
7	7	6	7
6	6	6	4
6	5	6	5
7	7	6	7
6	6	6	4
5	5	3	4
6	6	6	4
5	6	1	5
4	7	6	5
7	7	4	5
5	6	5	4

4	5	4	3
5	2	1	1
6	6	6	6
7	7	7	6
7	7	7	6
7	7	7	7
6	6	6	3
4	4	4	4
6	6	6	4
6	6	5	4
6	7	6	6
6	6	5	6
7	7	6	5
5	5	4	4
5	4	4	4
7	6	4	5
6	3	3	5
6	6	6	7
5	5	4	6
7	7	2	1
5	5	5	5
1	2	2	1
5	5	5	4
7	7	5	5
6	6	1	4
5	7	4	4
6	6	3	3
6	6	6	6
5	6	6	5
5	5	5	5
6	6	6	5
7	7	4	5
6	6	6	6
6	5	4	6
5	5	4	4
5	5	5	5
7	7	6	6
6	6	6	6
6	6	4	5
5	5	5	4
6	6	6	5
5	4	3	3
7	6	2	6
6	6	7	4
6	6	4	4
6	6	6	6
7	7	4	3
5	5	6	6
4	4	5	3

2	2	3	1
5	6	5	5
6	5	5	4
6	6	4	5
6	6	4	6
6			
6	6	6	5
5	5	5	4
6	6	6	4
5	5	4	4
5	6	5	5
5	5	1	5
5	5	4	
7	7	7	6
7	7	5	6
6	6	6	6
4	4	1	4
6	5	5	4
5	6	5	6
5	5	2	2
7	7	7	5
5	5	4	4
6	6	6	5
6	6	5	6
5	5	4	3
7	1	5	1
5	4	6	6
6	7	5	5
7	6	6	6
6	7	4	2
4	5	6	
6	6	6	5
5	6	4	6
6	5	3	4
7	6	5	6
7	7	7	5
6	6	7	3
7	7	7	7
3	3	3	1
6	5	6	4
6	6	6	6
1	1	1	1
7	7	7	7
4	4	5	4
4	4	4	4
6	7	5	4
6	5	5	4
6	6	2	2
5	6	5	5

6	6	1	4
7	7	5	6
7	6	7	4
3	2	1	3
7	7	5	4
5	5	2	2
6	7	4	3
7	7	6	6
6	6	6	6
7	7	5	6
7	7	5	6
6	6	4	3
7	5	6	5
5	5	3	4
7	7	7	6
6	7	4	6
6	6	5	5
6	6	6	6
5	5	4	3
4	5	5	4
7	7	7	7
5	6	4	3
3	3	2	2
5	4	4	4
5	5	5	4
5	5	6	4
7	7	7	7

15 - Verifica se são cur	16 - Demonstra empati	17 - Trabalha com infor	18 - Tem acesso a pes
6	6	5	7
5	4	5	4
2	6	6	4
4	5	6	7
7	6	7	6
3	6	6	6
4	4	6	6
3	5	5	4
3	5	5	5
4	5	5	1
6	6	4	5
5	6	6	2
6	7	6	6
6	6	6	6
5	5	6	7
3	4	5	3
5	7	6	7
6	6	6	7
5	7	6	6
2	5	5	2
6	6	7	7
5	5	5	3
6	6	4	5
6	7	4	7
7	7	7	6
4	7	7	4
4	7	7	6
5	6	6	6
6	6	6	5
6	6	6	6
6	5	6	6
2	7	6	6
6	6	7	5
6	7	6	6
3	4	3	3
6	7	7	7
5	7	7	4
7	7	7	7
7	7	7	7
7	7	7	7
3	4	3	3
5	6	7	4
6	6	7	6
7	7	7	6
7	7	7	6
4	4	4	1
6	5	6	6
4	5	5	5

5	6	6	6
4	5	6	5
4	7	7	6
3	6	5	3
6	7	7	7
4	5	4	2
5	6	5	6
6	6	6	6
6	6	6	5
6	7	6	6
6	6	7	6
5	5	7	5
6	5	6	7
5	7	6	7
6	6	5	5
7	6	7	5
5	5	6	4
5	7	7	4
5	7	7	5
5	5	5	5
5	6	5	3
5	6	6	1
5	7	6	6
4	5	5	4
5	7		5
5	7	6	5
6	6	6	3
4	4	4	3
6	6	6	4
4	4	7	4
4	5	6	3
6	6	6	5
6	7	5	6
4	3	3	3
6	6	6	6
6	6	6	6
3	5	3	4
5	6	6	6
5	6	6	6
5	6	6	6
5	5	5	6
6	7	7	6
5	7	6	5
5	5	5	5
3	6	6	4
6	7	6	6
5	5	5	5
6	6	6	7
6	6	6	4

5	7	7	2
6	7	6	6
5	6	7	6
7	7	7	4
4	7	5	3
4		6	2
2	4	6	5
5	5	7	6
6	7	7	7
6	7	6	6
7	7	7	6
7	7	7	7
7	7	7	4
7	7	7	7
4	4	4	4
6	7	7	7
5	6	6	7
6	6	6	6
7	7	7	7
5	5	5	5
7	6	7	6
4	6	6	6
6	6	7	7
6	7	6	5
6	7	6	6
6	7	6	3
1	3	1	2
3	4	6	5
6	6	7	6
5	5	7	2
6	7	7	5
1	1	1	1
5	7	7	4
6	6	5	7
4	4	4	4
1	4	4	4
6	6	5	4
2	7	7	7
5	6	5	5
1			1
7	7	7	2
1	6	4	2
6	7	7	7
4	5	5	3
4	6	6	5
4	5	6	5
1	4	4	3
5	6	6	6

6	6	6	6
4	7	6	2
6	7	7	7
7	6	7	7
5	7	7	6
7	7	7	7
3	4	7	6
7	6	7	7
5	5	5	4
6	6	6	4
6	7	7	7
5	7	7	3
7	7	7	7
7	7	6	4
6	7	4	7
3	5	5	5
5	5	7	5
5	7	5	6
3	5	6	2

6	6	5	6
6	6	6	5
4	6	6	6
7	6	7	7
7	7	7	3
6	7	6	6
4	6	7	5
5	7	6	7
6	7	7	7
2	4	6	1
6	5	6	6
4	5	4	3
6	6	6	4
4	6	6	6
5	6	6	4
5	7	7	4
6	7	7	6
5	6	6	5
6	5	6	6
4	6	6	5
5	5	5	5
7	6	7	7
5	6	6	7
4	5	6	6
5	6	6	3
5	5	5	6
5	6	5	5
6	5	7	4
5	5	6	6

4	6	6	6
2	1	2	1
6	6	6	6
7	7	7	7
7	6	7	7
7	7	7	7
5	5	5	6
5	6	3	4
4	6	4	7
4	6	6	5
7	6	6	4
6	6	6	6
6	7	7	6
4	5	5	6
4	5	5	3
4	5	5	4
3	6	6	6
6	6	7	6
6	6	5	4
1	4	4	2
5	5	5	6
1	1	7	6
4	6	6	4
6	7	6	6
5	7	6	2
5	6	6	7
4	6	6	3
6	7	6	6
6	6	6	6
5	5	5	5
5	5	6	5
6	6	7	4
6	6	6	4
2	6	6	6
5	6	5	6
5	6	7	5
6	7	6	7
6	6	6	6
5	6	4	4
5	5	4	5
7	5	6	7
5	5	4	4
6	6	7	2
6	7	6	5
6	7	7	4
6	6	6	6
6	7	6	6
6	5	6	6
4	4	5	5

1	4	4	4
5	6	6	7
6	6	6	6
5	5	5	4
6	5	6	4
6	7	6	5
4	4	5	4
5	6	6	7
4	5	5	4
5	7	6	2
6	5	6	1
3	5	5	5
6	7	6	6
6	7	7	7
5	6	7	6
4	1	5	4
4	6	5	4
6	5	4	5
2	5	5	5
6	7	7	7
4	5	5	4
6	6	7	7
6	6	5	2
5	6	6	4
1	1	7	4
6	7	6	7
7		7	6
6	7	7	6
4	7	7	7
5	7	6	6
6	6	6	6
5	5	5	6
4	6	6	4
6	5	7	6
5	6	7	5
6	1	7	7
	7		7
1	5	5	4
4	5	6	6
6	6	6	6
1	5	6	1
7	6	7	7
5	6	5	6
	5	4	4
4	6	6	6
5	4	6	6
3	3	6	4
5	5	5	5

7		7	3
7	7	7	5
5	6	7	7
2	7	5	2
6	7	6	6
5	6	4	4
6	6	7	7
5	7	7	6
5	6	6	5
7	7	7	6
7	7	7	7
4	5	6	1
5	6	7	5
5	5	5	5
6	7	7	7
6	7	7	4
5	6	6	6
6	7	7	7
3	5	4	4
4	4	5	4
6	6	6	7
5	6	6	4
2	2	3	3
5	5	4	5
5	6	5	5
5	6	5	6
7	7	7	7

19 - Clarifica objetivos (	20 - Trata as pessoas (	21 - Mantém as rédeas	22 - Resolve problemas
6	6	5	5
5	6	4	4
4	7	5	6
5	5	5	4
7	7	6	7
6	6	5	5
6	7	1	4
4	6	5	5
4	5	5	5
5	5	5	5
6	5	4	4
3	6	3	4
6	7	6	6
6	5	5	6
5	7	5	7
3	6	4	5
6	7	5	6
7	7	7	6
5	5	4	5
2	6	3	6
6	7	6	5
5	5	5	3
6	5	4	4
5	7	5	6
7	7	7	6
6	7	6	4
6	7	6	5
6	5	5	5
6	6	5	5
6	6	6	6
6	6	6	5
6	6	7	6
7	6	7	7
6	7	5	5
5	5	5	5
6	7	6	5
4	7	4	7
7	7	5	6
7	7	7	7
7	6	6	6
5	5	5	5
4	6	3	6
6	7	5	5
7	6	7	6
7	7	7	7
4	6	4	5
5	6	6	5
4	6	5	5

5	7	4	4
5	6	5	6
7	7	7	6
3	6	6	5
6	6	6	5
3	5	5	4
6	7	6	5
6	6	6	6
6	6	6	5
7	7	7	6
6	6	6	6
5	5	4	6
5	6	4	5
6	7	6	7
6	7	6	6
6	6	6	6
5	7	5	5
6	6	7	6
5	7	6	5
3	6	6	3
5	5	4	5
5	6	5	5
6	7	6	6
4	6	5	4
5	7	6	6
5	4	4	5
3	6	6	5
4	5	5	4
5	6	5	4
5	4	3	3
6	6	4	5
6	7	6	5
6	6	6	5
4	6	3	5
6	7	6	6
6	5	7	3
3	6	2	4
6	6	6	6
5	6	5	6
5	6	5	6
5	5	5	5
7	7	7	5
5	7	6	5
5	6	4	5
5	6	4	6
6	6	5	6
5	7	5	5
6	7	7	6
5	7	5	6

5	6	6	6
6	7	5	5
2	7	5	6
6	7	6	6
5	7	4	5
4	6	2	4
4	6	4	4
7	6	7	7
7	7	7	7
6	7	6	6
7	7	7	7
6	6	4	6
7	7	4	7
7	7	7	7
4	6	4	4
7	7	6	5
6	7	7	6
6	6	6	6
7	7	6	5
5	5	5	5
6	5	6	6
6	7	6	6
7	7	1	7
6	7	6	7
5	7	5	5
6	7	6	6
2	6	2	2
5	7	4	4
7	7	6	6

5	7	4	4
6	6	5	6
1	7	3	6
5	7	7	5
6	7	7	6
4	5	4	5
4	7	3	3
4	7	5	5
5	7	5	6
6	6	5	6
1	5	1	3
6	7	6	6
1	5	3	2
7	7	7	7
6	6	6	6
6	7	6	6
5	5	5	6
3	5	2	5
5	7	5	6

6	7	6	5
5	7	5	5
5	7	7	6
6	6	6	6
7	7	5	5
6	7	6	7
4	7	2	2
7	6	5	5
4	5	4	4
4	6	5	6
	7	7	7
6	7	7	5
7	7	6	5
6	7	3	5
6	5	7	6
4	6	4	5
5	5	5	5
5	6	5	5
2	6	4	4
4	7	6	5
5	6	6	4
4	7	5	6
7	6	6	6
7	7	4	6
5	7	5	7
5	6	5	6
6	6	6	5
6	6	6	6
2	6	6	5
6	7	6	6
4	7	5	4
6	7	1	6
6	7	6	6
5	6	5	5
5	7	5	6
6	6	6	5
5	6	5	5
6	6	7	7
5	7	6	6
4	6	5	5
7	7	7	6
4	7	7	7
5	7	4	4
5	6	6	6
5	4	5	5
4	7	4	4
6	7	6	6
5	5	6	5

6	7	5	6
2	7	1	5
6	7	7	7
6	7	6	7
7	7	7	7
7	7	7	7
5	7	5	7
5	7	4	4
5	6	4	6
5	7	6	5
6	7	6	7
6	6	4	6
7	7	6	7
5	6	5	4
4	5	4	4
6	7	5	6
5	6	4	6
6	6	6	6
4	6	6	5
2	7	4	5
5	5	5	5
6	7	6	6
5	6	6	6
6	7	6	6
4	7	5	3
5	6	5	5
3	5	4	4
5	7	5	6
6	7	6	6
5	5	5	5
5	5	5	5
7	6	6	6
6	6	4	5
6	6	4	6
5	6	5	5
5	6	6	5
6	7	5	6
6	7	7	6
5	6	6	4
5	5	5	5
6	7	7	5
4	5	5	5
6	7	6	5
6	7	5	5
6	7	6	5
6	6	5	6
7	7	4	5
5	5	5	6
4	5	4	4

1	6	3	4
6	7	6	6
5	7	5	7
5	5	5	5
6	6	5	5
6	7	4	6
4	4	4	4
5	7	6	4
4	4	4	4
6	7	5	5
4	6	4	5
5	5	4	5
7	7	7	6
7	7	7	6
6	7	6	6
3	4	1	4
4	6	4	5
5	5	4	4
4	5	2	5
6	7	6	7
5	5	5	5
6	6	6	6
5	6	5	6
6	6	4	4
6	7	1	6
5	6	5	5
7	7	7	6
6	7	6	6
7	7	4	7
6	7	5	5
6	7	6	6
5	7	5	6
5	6	6	5
5	7	6	6
5	6	7	7
6	6	7	4
7		7	7
2	7	2	3
5	6	6	5
6	6	6	6
5	5	1	1
7	6	5	7
5	6	6	6
4		4	4
5	7	5	5
6	6	5	6
4	4	3	3
4	6	5	4

6	7	4	6
7	7	6	6
6	7	6	7
2	6	5	3
7	7	6	6
5	7	2	6
7	6	5	7
6	7	7	6
5	6	6	5
6	7	7	7
7	7	2	6
5	7	6	6
6	6	5	6
5	5	4	4
7	7	7	7
5	7	4	6
6	6	6	6
6	7	6	6
5	6	5	5
4	6	6	5
7	7	7	7
6	6	6	5
2	4	3	3
5	7	5	5
5	5	5	5
5	7	7	6
7	7	7	7

23 - Impulsiona a unida	24 - Encoraja os subor	25 - Procura inovações	26 - Clarifica prioridade
6	6	6	6
4	4	5	5
4	4	5	5
5	5	4	5
7	7	6	7
5	5	5	6
4	1	4	4
4	5	5	5
6	5	5	5
4	5	5	4
4	5	5	5
6	6	6	6
6	7	6	7
6	6	7	7
5	6	7	7
4	4	3	4
6	6	6	6
7	6	6	6
4	5	3	4
6	4	4	4
6	6	7	6
5	5	5	5
4	5	5	5
5	6	6	5
7	6	6	6
6	7	6	6
5	7	6	6
5	6	6	6
5	6	6	6
6	6	6	6
6	5	6	6
6	6	5	6
7	7	7	7
5	5	5	5
6	5	3	5
6	6	4	5
4	5	6	6
5	6	7	7
7		7	7
7	7	7	7
6	5	3	5
5	6	5	5
5	5	5	5
7	7	6	6
7	7	7	7
4	4	4	4
5	5	5	5
5	3	4	4

6	5	5	6
6	5	6	5
6	6	5	6
6	4	4	4
6	7	6	6
4	3	2	4
6	6	6	5
6	6	6	6
5	5	5	5
7	6	5	5
6	6	6	6
6	5	6	6
5	6	5	5
7	7	7	6
6	6	6	6
7	6	7	6
4	6	4	5
6	6	5	5
5	2	3	2
3	5	4	4
5	5	5	5
5	3	5	5
6	7	6	6
4	4	4	4
6	5	4	4
5	6	6	6
5	5	4	4
4	4	4	4
5	4	4	5
6	3	6	6
5	6	5	6
6	5	5	5
6	6	6	6
5	4	4	4
6	6	6	6
6	5	6	6
2	4	4	2
6	6	6	6
5	5	5	5
6	5	5	6
5	5	5	5
6	7	6	7
5	6	6	6
6	6	6	6
6	4	6	4
6	6	6	6
5	4	5	5
6	6	6	6
6	6	5	7

6	6	6	7
6	7	7	6
5	5	6	5
6	6	6	6
6	5	6	6
4	4	4	3
5	3	4	3
7	6	7	7
7	6	6	7
6	5	6	6
7	7	7	7
6	7	6	6
7	7	7	7
7	7	7	7
4	4	4	4
6	7	6	6
	5		5
6	6	6	6
7	7	7	7
5	5	5	5
7	6	7	7
6	6	6	6
7	7	7	7
7	7	7	7
5		5	5
5	6	6	5
4	4	4	4
4	4	5	5
6	6	6	6
5	5	6	6
5	7	7	7
5	4	5	5
5	7	5	5
6	7	6	6
4	5	5	5
6	1	3	3
6	4	6	6
6	6	6	7
6	6	6	6
	4	4	3
6	6	6	6
2	4	3	4
7	7	7	7
6	5	4	5
6	6	6	6
5	5	5	5
5		2	2
5	5	7	7

6	6	6	6
5	5	5	5
6	6	6	5
7	7	7	7
5	7	7	7
7	7	7	7
6	7	7	7
7	6	6	6
5	5	4	4
6	6	6	6
7	7	7	7
5	6	6	6
5	7	7	7
7	6	7	7
6	6	5	7
4	4	5	4
5	5	6	5
5	6	5	6
3	5	2	6

6	5	5	5
6	4	4	5
5	6	6	6
6	7	7	7
7	7	7	7
6	5	5	6
4	6	4	6
5	5	6	6
6	6	7	7
3	4	4	4
6	7	6	6
5	5	4	5
6	6	6	6
6	7	6	7
5	5	5	5
7	7	7	6
6	6	6	6
4	6	5	5
6	6	6	6
6	6	6	6
5	6	5	5
6	6	6	7
6	6	6	6
4	4	4	5
6	5	5	5
5	5	5	5
6	5	7	6
6	7	7	7
5	5	4	5

5	5	4	5
3	1	4	4
7	6	7	6
7	7	7	6
7	7	7	7
7	7	7	7
6	5	6	7
5	5	5	6
5	6	6	6
5	5	5	5
7	6	7	7
6	6	6	6
6	7	7	7
5	5	5	5
4	5	4	5
5	5	5	5
4	3	5	5
6	6	6	6
5	5	5	5
7	7	7	7
5	5	5	5
6	4	6	6
5	6	6	5
6	7	6	6
5	3	3	3
5	6	6	5
4	6	5	6
6	6	6	6
6	6	6	6
5	5	5	5
5	6	6	6
6	6	6	7
5	6	6	6
6	6	6	6
5	4	5	4
5	5	5	5
6	7	7	7
6	6	6	6
5	6	6	6
5	5	5	5
6	6	5	6
5	4	5	5
6	7	7	6
5	6	5	6
7	6	5	5
6	6	6	6
7	7	7	6
5	5	4	4
5	5	5	5

1	1	5	5
6	6	6	6
7	6	4	4
5	5	5	5
5	5	6	6
6	6	6	6
4	5	4	4
6	5	6	5
4	5	5	5
6	6	5	5
5	5	5	4
5	5	5	5
6	6	6	6
6	6	6	6
6	6	6	6
4	5	4	4
5	5	5	5
4	5	5	5
4	5	5	4
6	7	7	7
5	5	5	5
5	5	7	6
6	5	6	6
6	6	6	6
7	1	7	5
4	5	5	6
6	6	6	6
6	7	6	6
7	7	7	7
5	3	5	6
6	6	6	6
6	6	6	6
6	6	5	5
6	6	6	6
6	6	6	6
6	1	6	6
	7		7
3	4	2	2
5	6	6	5
6	6	6	6
1	1	1	1
7	7	7	6
6	5	5	5
		4	
6	6	6	6
5	6		6
3	6	4	5
5	5	4	4

6	4	6	6
7	7	7	7
6	7	7	6
3	3	5	3
7	7	7	6
5	5	5	5
7	6	7	7
6	7	7	7
6	6	6	6
7	7	6	7
7	7	7	7
6	1	5	5
7	7	6	6
5	5	5	5
7	7	7	7
5	6	6	6
5	6	6	6
6	6	6	6
5	5	5	6
4	4	4	5
7	7	7	7
6	5	6	6
3	2	3	2
5	5	5	5
5	5	5	4
7	7	6	6
7	7	7	7

27 - Apresenta aos sup	28 - Traz à unidade um	29 - Preocupa-se com a	30 - Realça a obtenção
6	6	6	6
4	5	4	4
5	5	5	5
5	5	5	4
6	7	6	6
6	5	6	6
4	3	4	2
4	5	6	5
4	6	5	5
4	5	5	1
6	4	4	4
6	4	6	5
6	6	7	7
6	6	7	7
7	6	6	5
4	4	4	4
6	6	6	6
7	6	7	6
5	4	6	5
3	2	4	3
7	6	7	6
5	7	5	4
6	4	4	4
7	6	7	5
6	7	6	6
4	6	7	6
6	4	7	6
6	6	6	6
6	5	6	6
6	5	5	6
6	6	6	6
6	6	6	6
6	6	7	6
5	5	5	5
5	5	4	4
4	6	5	5
6	6	7	6
7	6	6	6
7	7	7	7
7	7	6	7
5	5	4	4
6	6	5	6
6	5	5	5
7	6	7	7
6	7	7	7
4	4	4	4
5	6	5	5
4	5	5	5

4	5	6	5
7	6	5	5
5	6	7	6
4	5	6	6
6	6	6	6
3	5	4	4
5	6	6	6
6	6	6	6
5	6	6	5
6	7	7	6
5	4	6	6
6	5	4	5
4	4	5	
7	6	7	6
6	6	6	6
6	6	7	6
3	4	6	4
6	7	5	5
4	6	6	4
4	4	4	4
4	5	5	5
1	1	2	4
6	6	7	5
4	6	6	4
5	5	5	5
6	6	6	5
5	6	6	6
4	4	4	4
4	5	5	5
4	6	3	4
5	6	6	5
5	7	7	6
5	7	7	6
3	3	3	4
6	6	6	6
5	6	6	6
2	4	4	4
6	6	6	6
5	5	6	5
6	6	6	5
6	5	5	5
6	6	7	6
6	6	6	5
6	6	7	6
5	5	4	4
6	6	7	6
5	5		5
5	6	6	5
5	6	5	5

4	6	5	5
6	6	6	6
7	7	6	6
5	4	7	5
5	6	7	5
5	5	3	5
5	4	6	3
7	7	7	7
6	6	7	6
4	6	6	6
7	7	7	6
6	6	6	7
7	7	7	7
7	7	7	7
6	4	3	5
6	5		6
6		5	6
6	6	6	6
6	7	7	7
5	7	5	5
6	5	6	6
6	6	6	4
7	7	7	7
6	6	7	6
5	6	6	5
6	6	7	5
4	4	3	3
4	5	5	5
6	6	7	6
4	5	5	5
5	6	7	5
3	5	1	3
4	6	7	5
7	7	7	5
5	4	4	4
3	7	3	4
6	7	6	6
6	6	6	6
6	5	6	7
3	2	2	2
5	6	7	6
1	1	5	1
7	7	7	7
4	6	5	5
6	6	6	6
6	4	5	5
2	6	6	3
6	5	6	6

6	6	7	6
5	6	6	3
5	7	7	6
6	6	7	6
6	6	7	7
7	7	7	6
7	4	7	6
4	6	6	6
4	4	5	5
6	5	5	5
7	7	7	7
6	5	7	5
5	6	7	6
6	5	7	4
7	7	7	6
4	4	4	4
5	5	5	4
5	5	6	6
2	3	4	2
4	5	5	5
4	5	5	4
5	6	6	5
6	7	7	7
7	6	7	7
6	6	7	6
3	7	7	6
5	7	7	6
6	6	7	6
2	4	4	3
6	6	5	6
4	5	5	4
4	6	7	6
6	6	6	5
5	5	5	5
5	6	7	6
6	5	6	6
6	5	5	5
7	6	6	6
6	6	7	6
5	6	6	5
6	7	7	
6	7	7	6
4	4	4	3
5	6	6	5
6	5	6	5
6	7	5	6
6	6	6	6
4	5	5	5

4	6	6	5
4	4	2	4
6	7	6	6
7	6	7	6
4	5	7	7
7	7	7	7
6	6	5	5
5	6	5	4
7	7	6	5
5	6	5	5
5	7	7	6
6	6	6	6
6	6	7	7
5	5	6	5
4	3	5	4
4	5	5	5
4	5	4	4
6	6	7	6
4	4	6	4
6	6	4	4
5	5	5	5
5	6	2	2
5	6	6	5
5	6	7	7
1	3	7	3
5	6	6	6
3	4	6	5
6	6	7	6
6	6	5	6
5	5	5	5
5	6	5	5
7	7	7	7
6	4	6	5
6	6	6	6
5	5	5	5
5	6	5	1
6	6	7	7
6	6	6	6
6	6	6	5
5	5	5	5
6	6	6	6
4	5	5	4
5	5	7	6
6	6	7	6
6	5	7	5
6	5	6	5
6	6	7	6
6	5	5	5
5	5	4	4

4	1	2	2
6	6	6	6
5	5	7	5
5	5	5	5
6	5	5	6
5	6	6	5
4	4	4	4
6	6	6	6
6	4	4	4
4	6	7	7
4	5	5	4
5		5	5
7	7	7	7
6	6	7	6
6	6	7	6
3	2	1	2
6	5	5	5
4	4	5	4
4	5	5	5
7	6	7	7
4	5	5	5
7	5	6	6
6	5	3	5
4	5	6	5
6	7	1	1
7	6	7	5
5	6	6	5
6	6	7	6
6	7	7	6
4	5	6	5
5	5	6	6
4	5	5	5
4	6	5	5
6	5	5	5
6	7	6	5
5	6	1	1
7		7	
3	4	6	2
5	6	6	6
6	6	6	6
1	1	1	1
6	7	7	7
5	5	6	4
4	5	4	4
6	6	7	6
6	5	4	
5	5	5	5
4	5	6	5

3	6	6	6
6	7	7	7
6	7	7	6
2	6	7	3
6	7	7	6
5	5	5	2

6	7	7	6
7	7	7	7
5	5	5	6
7	7	7	6
6	6	7	7
5	6	5	6
7	7	6	6
4	4	5	5
7	7	7	7
4	5	7	6
6	6	6	5
6	6	7	6
5	5	4	4
5	5	5	4
7	7	7	6
5	6	6	6
2	3	3	2
5	5	4	4
5	5	6	5
6	7	7	6
7	7	7	7

31 - Cria uma equipa d	32 - Analisa planos e projetos:
6	6
4	4
5	5
5	5
6	7
1	4
1	1
5	5
2	3
1	1
2	6
6	6
7	6
5	6
3	3
3	4
6	5
7	5
5	2
3	2
7	6
5	5
2	6
7	5
6	5
3	4
2	5
5	5
2	6
6	5
4	6
4	2
6	7
4	5
5	4
6	4
4	4
7	7
7	7
7	7
5	4
1	5
4	4
6	7
7	7
4	4
1	6
4	3

4	4
5	5
4	4
4	3
6	6
4	4
6	5
6	6
5	5
4	6
6	6
5	5
5	2
7	6
6	6
6	6
3	4
6	6
1	1
3	6
5	5
5	5
4	5
4	4
3	4
5	5
3	5
4	4
4	5
2	5
4	6
4	5
6	6
3	3
7	6
6	6
1	1
6	6
5	4
6	5
6	6
5	4
5	4
1	4
5	5
5	5
5	4
5	5

4	4
7	6
6	6
5	6
5	5
1	5
2	1
7	
5	6
5	6
	7
7	7
5	7
7	6
6	6
5	5
	6
6	6
7	6
5	5
6	6
5	4
7	7
7	6
3	5
5	4
2	2
5	5
6	7
5	5
7	6
1	1
5	5
6	6
4	4
1	1
4	4
6	6
7	7
1	1
6	6
1	1
7	7
6	5
6	3
5	5
1	1
6	6

5	5
5	4
7	6
6	6
5	6
6	7
4	3
5	6
5	5
5	5
5	5
3	6
7	7
4	4
5	5
2	3
2	5
5	5
2	2
1	4
5	5
4	4
7	7
7	7
5	5
4	3
7	5
4	6
3	4
5	5
5	4
6	6
5	4
4	4
6	6
6	6
3	3
6	6
4	
5	5
6	6
6	4
3	3
4	5
5	5
6	6
4	4
4	5

4	4
1	4
7	7
7	7
4	7
7	7
5	7
5	5
5	4
5	3
7	6
4	6
6	4
5	5
5	4
4	4
2	5
6	6
4	5
1	1
5	4
6	5
3	3
6	5
5	4
6	6
5	6
6	6
5	6
5	5
5	5
7	7
4	3
6	5
4	5
5	1
6	7
6	6
4	4
5	5
6	5
1	5
7	6
	5
4	4
5	6
6	6
4	6
4	4

2	2
5	6
1	1
3	5
5	6
4	5
4	4
5	4
4	4
5	5
1	4
5	4
6	6
7	6
6	4
2	2
6	5
4	4
2	2
6	6
4	5
1	2
4	5
1	6
1	5
5	5
7	7
5	6
4	4
3	3
6	5
5	5
4	4
5	6
7	6
1	6
7	7
4	4
4	4
6	6
1	1
7	7
6	5
4	4
4	4
6	6
5	5
4	5

4	4
6	7
7	6
2	2
4	6
2	2
6	4
7	5
6	6
4	5
6	7
7	6
5	4
5	5
7	7
4	2
6	5
7	6
4	4
4	3
7	7
6	5
2	2
5	4
5	4
5	4
7	7
